# A Pointwise Method for Identifying Biomechanical Heterogeneity of the Human Gallbladder

**DOI:** 10.3389/fphys.2017.00176

**Published:** 2017-03-31

**Authors:** Wenguang Li, Nigel C. Bird, Xiaoyu Luo

**Affiliations:** ^1^School of Engineering, University of GlasgowGlasgow, UK; ^2^Academic Surgical Unit, Royal Hallamshire HospitalSheffield, UK; ^3^School of Mathematics and Statistics, University of GlasgowGlasgow, UK

**Keywords:** gallbladder, strain energy function, heterogeneity, anisotropic property, constitutive law, optimization, inverse problem

## Abstract

Identifying the heterogeneous biomechanical property of human gallbladder (GB) walls from non-invasive measurements can have clinical significance in patient-specific modeling and acalculous biliary pain diagnosis. In this article, a pointwise method was proposed to measure the heterogeneity of ten samples of human GB during refilling. Three different points, two on the equator of GB body 90° apart and one on the apex of GB fundus, were chosen to represent the typical regions of interest. The stretches at these points were estimated from ultrasound images of the GB during the bile emptying phase based on an analytical model. The model was validated against the experimental data of a lamb GB. The material parameters at the different points were determined inversely by making use of a structure-based anisotropic constitutive model. This anisotropic model yielded much better accuracy when compared to a number of phenomenologically-based constitutive laws, as demonstrated by its significantly reduced least-square errors in stress curve fitting. The results confirmed that the human GB wall material was heterogeneous, particularly toward the apex region. Our study also suggested that non-uniform wall thickness of the GB was important in determining the material parameters, in particular, on the parameters associated with the properties of the matrix and the longitudinal fibers—the difference could be as large as 20–30% compared to that of the uniform thickness model.

## Introduction

Human gallbladder (GB) is a small pear-shaped organ that is attached to the underside of the right lobe of the liver. Its function is to store and concentrate bile produced continuously by the liver. Induced by cholecystokinin (CCK), bile can be expelled from the GB to the gut to aid the digestion of fat. Cholecystitis, often due to blockage of the cystic duct by gallstones, and acalculous biliary pain are common GB diseases that affect both women and men (Cozzolino et al., 1963; Williamson, 1988). The symptoms in acalculous biliary pain disease vary widely from discomfort to severe pain, which usually follows food intake. However, the painful symptoms remain in nearly 50% patients following gallbladder removal (Cholecystectomy) (Smythe et al., 1998, 2004). This is in part due to the lack of understanding of the underlying mechanism for acalculous biliary pain.

Interestingly, many human tissues, such as artery, breast, liver, and pancreas, can develop local disease, examples include vulnerable plaque (Baldewsing et al., 2004a; Trivedi et al., 2007), atheroma in coronary and femoral arteries (Chandran et al., 2003; Baldewsing et al., 2004b; Hamilton et al., 2005), arterial stenosis (Franquet et al., 2011), and cerebral aneurysms (Zhao et al., 2011a,b). Biomechanical properties of the diseased soft tissue are different to those of the healthy ones and are often heterogeneous. Inverse methods have been developed to identify isotropic biomechanical properties (Chandran et al., 2003; Baldewsing et al., 2004a,b; Hamilton et al., 2005; Trivedi et al., 2007; Franquet et al., 2011) in terms of the Young's modulus. In studies by Zhao, Raghavan, and Lu, pointwise inverse approaches were used to reveal the anisotropic heterogeneous biomechanical properties of cerebral aneurysms (Zhao et al., 2011a,b), ascending thoracic aneurysms (Davis et al., 2015) and murine aortas (Bersi et al., 2016) on a membrane mechanic model.

Healthy human GB wall is commonly regarded as a homogenous anisotropic non-linear material in passive state, i.e., bile refilling phase (Li et al., 2012, 2013; Xiong et al., 2013). However, recent work based on *in vitro* test of a healthy lamb GB suggested that this might not be true (Genovese et al., 2014). In addition, human acalculous biliary disease can lead to increased material heterogeneity in the GB wall. In this paper, we addressed this issue by extending the homogenous anisotropic non-linear biomechanical model for human GB wall proposed in Li et al. (2013) to a heterogeneous anisotropic case. We used an inverse pointwise method to identify the heterogeneous anisotropic property at three different points on the GB wall. The method was based on an ellipsoid membrane model and an in-house developed program using MATLAB.

## Computational models

### Geometrical model and stresses under internal pressure

**Figure 1:**
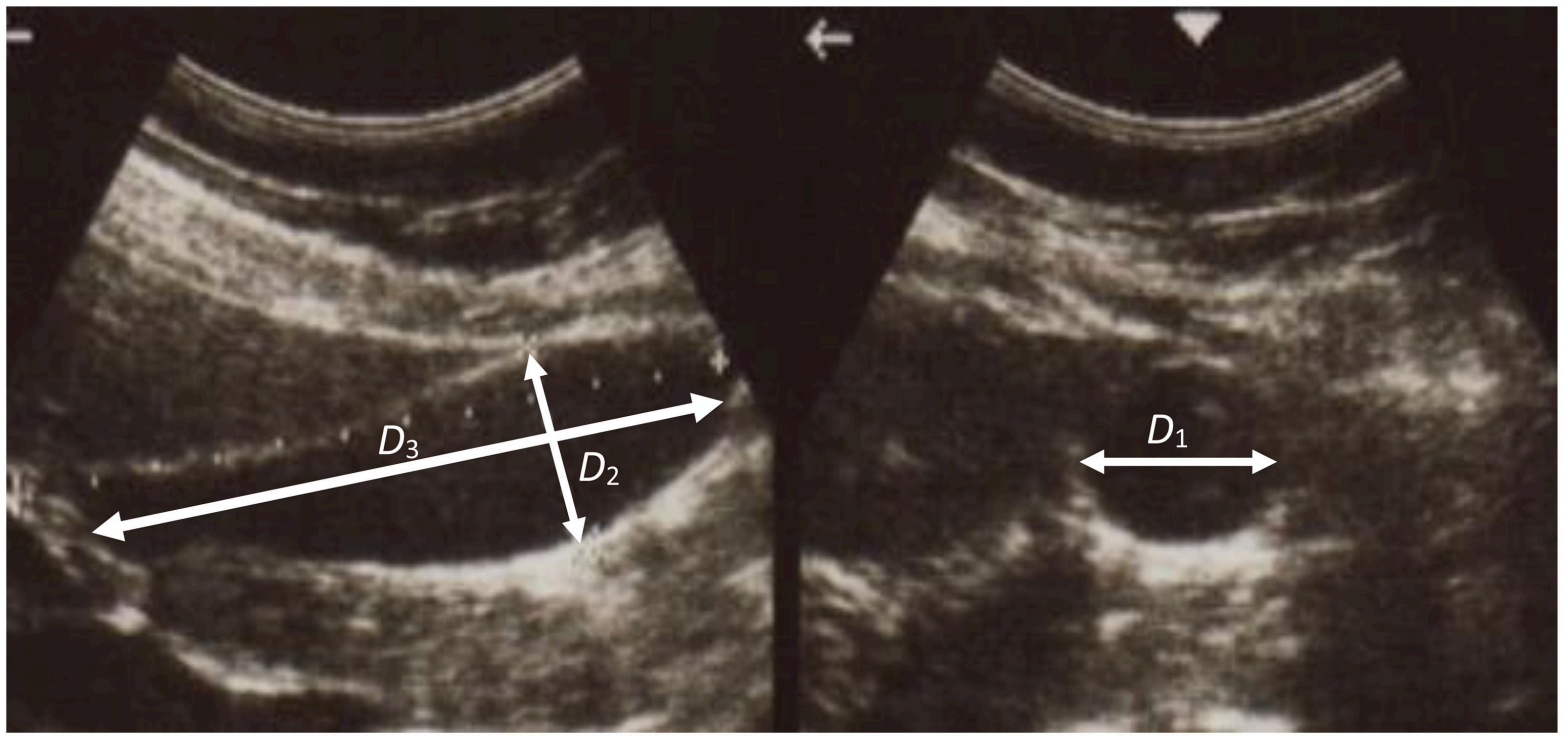
**A typical ultrasonic image of human gallbladder during the emptying phase**.

**Figure 2:**
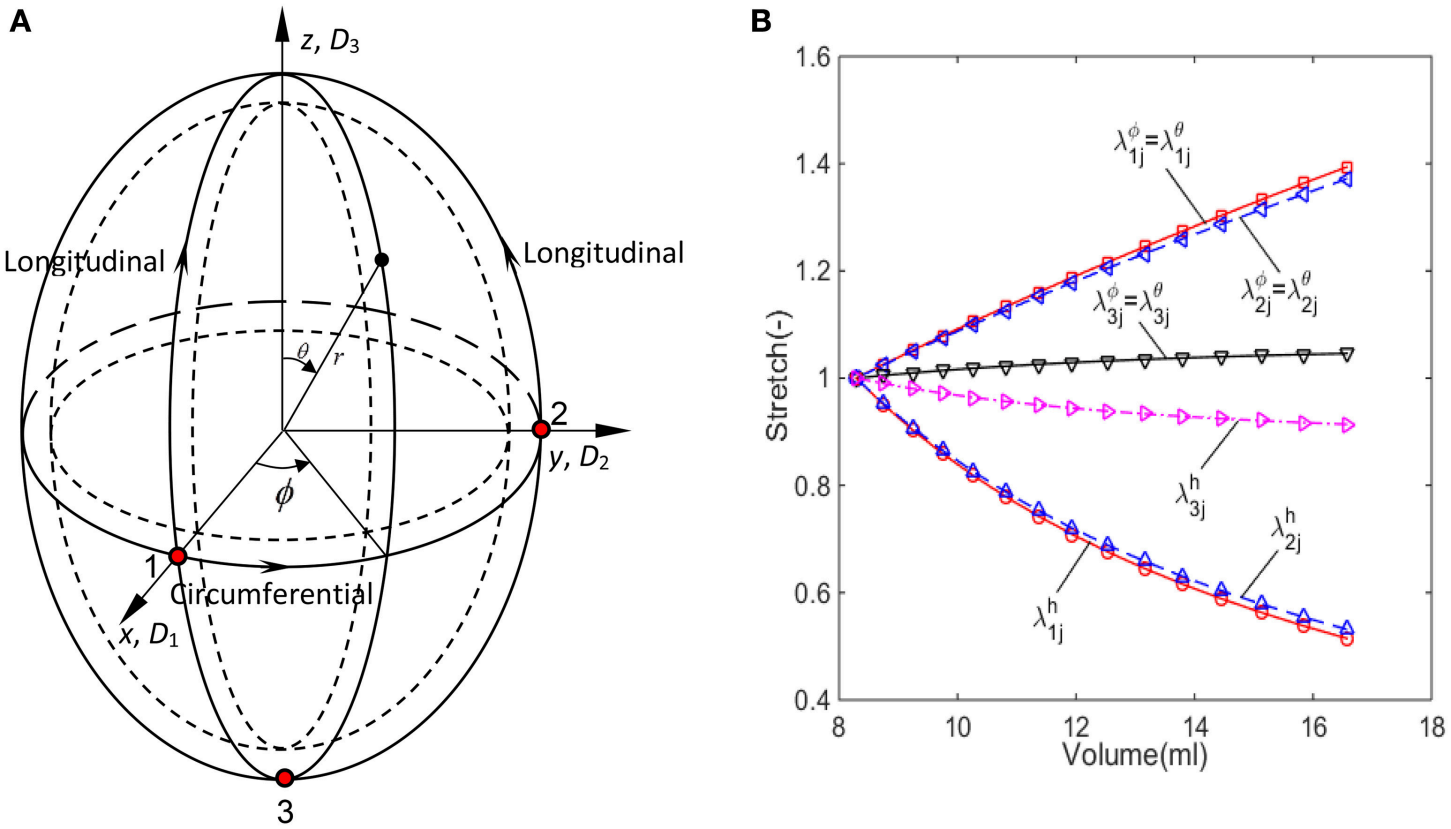
**The imaged-based ellipsoid model for GB during the refilling phase, (A)** ellipsoid model with three control points on the surface, **(B)** the stretch components estimated from the ellipsoid model for GB sample No.1 listed in Table 1.

A series of ultrasonic images of acalculous human GB had been scanned in 10 min interval for 60 min during the emptying phase at the Sheffield Hallamshire Teaching Hospital. A typical example is illustrated in Figure 1, marked by the three axes *D*_1_, *D*_2_, and *D*_3_ (*D*_1_ ≤ *D*_2_ ≤ *D*_3_). From these images we generated the corresponding ellipsoid models, as shown in Figure 2A, which are used to estimate the GB volume.

The passive biomechanical property of GB wall exhibits in the refilling phase only, hence we will focus this process. The refilling phase is the reverse process of the emptying phase. This means that for the same volume, the refilling and the emptying phases share the same ellipsoid (Li et al., 2013). The heterogeneous anisotropic biomechanical property of human GB wall in the refilling phase will be determined inversely at points 1, 2, and 3 in a GB wall. Point 1 is an intersected point of two ellipses, one is along the equator and the other is in the longitudinal direction in a meridian plane, point 2 is also on the equator but 90° apart from point 1, and point 3 is at the apex as shown in Figure 2A. In the spherical coordinate system (*r*, ϕ, θ), the coordinates of points 1, 2, and 3 are (*D*_1_/2, 0, π/2), (*D*_2_/2, π/2, π/2), and (*D*_3_/2, 0, π), respectively.

Observing that the volume of the GB model was reduced by 50% by the end of emptying (Li et al., 2013), we chose the end of the emptying configuration of the GB as the reference configuration.

We interpolated the GB model with fifteen time moments throughout the refilling phase based on GB images using the geometrical similarity (Li et al., 2011). The GB wall circumferential and longitudinal in-plane stretches at point 1 at a time instant *t*_*j*_ is calculated with (Ragab and Bayoumi, 1998).

(1) $$\{\begin{array}{l} \lambda_{1j}^{\phi }=1+\frac{u_{r}}{D_{11}/2\;}+\frac{D_{11}}{2}\frac{\partial u_{\phi }}{\partial \phi }\\ \lambda_{1j}^{\theta }=1+\frac{u_{r}}{D_{11}/2\;}+\frac{D_{11}}{2}\frac{\partial u_{\theta }}{\partial \theta } \end{array}$$

where the radial displacement *u*_*r*_ = 0.5 (*D*_1*j*_ − *D*_11_), and *j* = 1,2,3…,*N*, here *N* = 15, *D*_11_ is the length of the principal axis *D*_1_ at time *t*_1_, *D*_1*j*_ is the length of the principal axis *D*_1_ at time *t*_*j*_. Since point 1 is on the axis of the ellipsoid, symmetry requires that ∂*u*_ϕ_/∂ϕ = ∂*u*_θ_/∂θ = 0. Hence, Equation (1) can be simplified to:

(2) $$\{\begin{array}{l} \lambda_{1j}^{\phi }=1+\frac{0.5\left(D_{1j}-D_{11}\right)}{0.5D_{11}}=D_{1j}/D_{11}\;\\ \lambda_{1j}^{\theta }=1+\frac{0.5\left(D_{1j}-D_{11}\right)}{0.5D_{11}}=D_{1j}/D_{11}\; \end{array}$$

The incompressibility of the GB wall means that the stretch component of GB thickness must satisfy:

(3) $$\lambda_{1j}^{h}=1/\left(\lambda_{1j}^{\phi }\lambda_{1j}^{\theta }\right)\;.$$

Similarly, the stretch components are for point 2:

(4) $$\{\begin{array}{l} \lambda_{2j}^{\phi }=\lambda_{2j}^{\theta }=D_{2j}/D_{21}\;\\ \lambda_{2j}^{h}=1/\left(\lambda_{2j}^{\phi }\lambda_{2j}^{\theta }\right)\;, \end{array}$$

and for point 3:

(5) $$\{\begin{array}{l} \lambda_{3j}^{\phi }=\lambda_{3j}^{\theta }=D_{3j}/D_{31}\;\\ \lambda_{3j}^{h}=1/\left(\lambda_{3j}^{\phi }\lambda_{3j}^{\theta }\right)\;. \end{array}$$

where *D*_21_ and *D*_2*j*_ are the length of principal axis *D*_2_ at time *t*_1_ and *t*_*j*_, while *D*_31_ and *D*_3*j*_ are the length of principal axis *D*_3_ at time *t*_1_ and *t*_*j*_.

These stretch components at *t*_*j*_ (*j* = 1,…,*N*) and point *i* (*i* = 1,2,3) can be presented simply:

(6) $$\{\begin{array}{l} \lambda_{ij}^{\phi }=\lambda_{ij}^{\theta }=D_{ij}/D_{i1}\;\\ \lambda_{ij}^{h}=1/\left(\lambda_{ij}^{\phi }\lambda_{ij}^{\theta }\right)\;. \end{array}$$

The stretches during the refilling phase are plotted against the GB volume in Figure 2B at points 1, 2, and 3 for a typical GB sample. The GB volume changed with time exponentially based on an earlier model in Li et al. (2011): *V* = *G*e^*Ht*^ + *M*, where *G*, *H*, and *M* are parameters determined analytically using the measured GB volume and pressure at the moments *t*_1_ and *t*_*N*_.

The expressions of in-plane stress components in the GB wall during the refilling phase were the same as these in the emptying phase (Li et al., 2011) since we assumed the GB material is an elastic membrane:

(7) $$\{\begin{array}{l} \sigma_{ij}^{\theta }=p_{j}F_{\theta }F_{n}\\ \sigma_{ij}^{\phi }=p_{j}\frac{F_{\phi }}{F_{n}}, \end{array}$$

where *p*_*j*_ is the refilling pressure at time *t*_*j*_, and *F*_θ_, *F*_ϕ_, and *F*_*n*_ are the functions describing the geometry of the GB:

(8) $$\{\begin{array}{l} F_{\theta }=\frac{D_{3j}K_{1j}K_{2j}}{4h_{ij}}\left(1-\frac{K_{1j}^{2}-K_{2j}^{2}}{K_{1j}^{2}K_{2j}^{2}}\cos\;2\phi_{i}\right)\\ F_{\phi }=\frac{D_{3j}}{4K_{1j}K_{2j}h_{ij}}\left[\begin{array}{l} K_{1j}^{2}K_{2j}^{2}+\left(K_{1j}^{2}+K_{2j}^{2}-2K_{1j}^{2}K_{2j}^{2}\right)\sin^{2}\theta_{i}\\ +\;\left(K_{1j}^{2}-K_{2j}^{2}\right)\cos^{2}\theta_{i}\cos\;2\phi_{i} \end{array}\right]\\ F_{n}=\frac{\sqrt{K_{1j}^{2}\;\cos^{2}\;\theta_{i}\;\cos^{2}\;\phi_{i}\;+\;K_{2j}^{2}\;\cos^{2}\;\theta_{i}\;\sin^{2}\;\phi_{i}\;+\;\sin^{2}\;\theta_{i}}}{\sqrt{K_{1j}^{2}\;\sin^{2}\;\phi_{i}\;+\;K_{2j}^{2}\;\cos^{2}\;\phi_{i}}} \end{array}$$

where *K*_1*j*_ = *D*_1*j*_/*D*_3*j*_, *K*_2*j*_ = *D*_2*j*_/*D*_3*j*_, *h*_*ij*_ is the GB wall thickness at point *i*, and time *t*_*j*_, and $D_{ij}=\lambda_{ij}^{\theta }D_{i1}$, $h_{ij}=\lambda_{ij}^{h}h_{i1}$. The internal pressure *p*_*j*_ is given by Li et al. (2013):

(9) $$p_{j}=p_{1}{\left(\frac{p_{N}}{p_{1}}\right)}^{t_{j}/t_{N}\;},t_{j}\in \left[0,t_{N}\right]$$

where *p*_*N*_ is the mean final bile pressure in a GB after the refilling phase chosen to be 1,466.5 Pa (11 mmHg), *p*_1_ is the bile pressure when the refilling starts, *p*_1_ = 466.6 Pa (3 mmHg), and *t*_*N*_ is the total time of the refilling phase. These values are estimated from *in vivo* measurements (Li et al., 2013).

### The constitutive model

**Figure 3:**
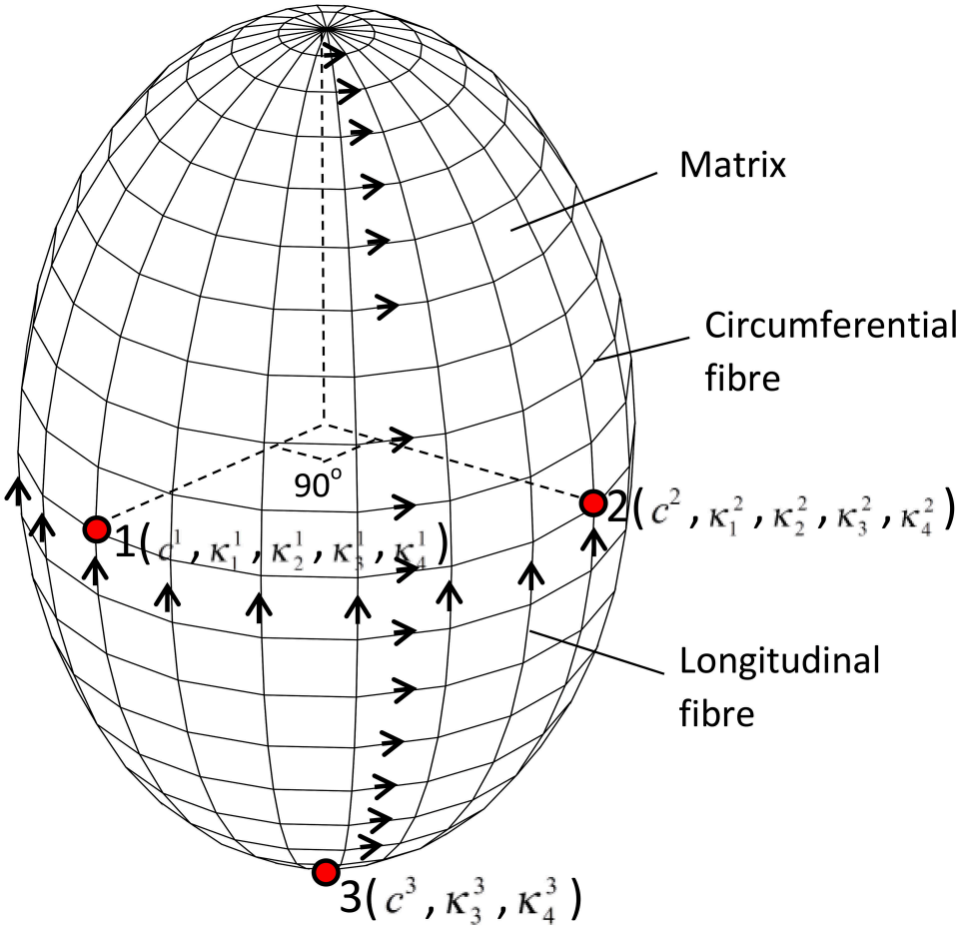
**The GB wall is composed of matrix and two families of fibers, the thirteen material parameters are location dependent, changing from points 1–3**.

To determine the heterogeneous material parameters of human GB wall, the structure-based anisotropic constitutive model used in Li et al. (2013) was extended so that the material parameters are location dependent. At each point, the GB wall is assumed to be composed of homogeneous matrix and two families of fibers along the circumferential and longitudinal directions, respectively, as shown in Figure 3. The strain energy functions are:

(10) $$\begin{array}{l} \psi_{i}=c^{i}\left(I_{1}-3\right)+\frac{\kappa_{1}^{i}}{2\kappa_{2}^{i}}\left[e^{\kappa_{2}^{i}{\left({\left(\lambda_{i}^{\phi }\right)}^{2}-1\right)}^{2}}-1\right]\\ \;+\;\frac{\kappa_{3}^{i}}{2\kappa_{4}^{i}}\left[e^{\kappa_{4}^{i}{\left({\left(\lambda_{i}^{\theta }\right)}^{2}-1\right)}^{2}}-1\right]. \end{array}$$

where the parameters *c*^*i*^, $\kappa_{m}^{i}$ (*i* = 1,2,3, *m* = 1–4) are location dependent. The total number of material property constants in Equation (10) for points 1–3 should be 15 in general. However, at point 3, there are no circumferential fibers, so the second term in Equation (10) disappears, i.e., $\kappa_{1}^{3}$ and $\kappa_{2}^{3}$ vanish. Hence, there are a total of 13 parameters to be determined.

The in-plane Cauchy stress components at *t*_*j*_ are:

(11) $$\{\begin{array}{l} \sigma_{ij}^{\prime \phi }=2c^{i}\left(\lambda_{ij}^{\phi 2}-\lambda_{ij}^{h2}\right)+2\lambda_{ij}^{\phi 2}\kappa_{1}^{i}\left(\lambda_{ij}^{\phi 2}-1\right)\;\exp\;\left(\kappa_{2}^{i}{\left(\lambda_{ij}^{\phi 2}-1\right)}^{2}\right)\\ +\;\sigma_{i1}^{\phi }\\ \sigma_{ij}^{\prime \theta }=2c^{i}\left(\lambda_{ij}^{\theta 2}-\lambda_{ij}^{h2}\right)+2\lambda_{ij}^{\theta 2}\kappa_{3}^{i}\left(\lambda_{ij}^{\theta 2}-1\right)\;\exp\;\left(\kappa_{4}^{i}{\left(\lambda_{ij}^{\theta 2}-1\right)}^{2}\right)\\ +\;\sigma_{i1}^{\theta } \end{array}.$$

where $\sigma_{i1}^{\phi }$ and $\sigma_{i1}^{\theta }$ (*i* = 1,2,3) are interpreted as the initial stresses imbedded in the GB wall, which are estimated using Equations (7–9) with the internal pressure *p*_1_.

### Comparison with other constitutive models

Several phenomenological anisotropic strain energy functions have been proposed for soft tissues. Here we did not intend to be exhaustive but will choose three commonly used strain energy functions for comparisons. These include the Fung strain energy function (Ferruzzi et al., 2011):

(12) $$\begin{array}{l} \psi =\frac{c}{2}\;\{e^{\frac{1}{4}\;[a_{1}\;{\left(\lambda^{\phi 2}\;-1\;\right)}^{2}\;+a_{2}{\left(\lambda^{\theta 2}\;1\right)}^{2}}\\ \;{\;}^{+2a_{3}\left(\lambda^{\phi 2}\;-\;1\right)\left(\lambda^{\theta 2}\;-\;1\right)]}-1\}\;, \end{array}$$

The Choi-Vito strain energy function (Ferruzzi et al., 2011):

(13) $$\begin{array}{c} \begin{array}{l} \psi =c\;\{e^{\frac{1}{4}a_{1}{\left(\lambda^{\phi 2}\;-\;1\right)}^{2}\;+\;a_{2}{\left(\lambda^{\phi 2}\;-\;1\right)}^{2}}\\ \;+\;e^{\frac{1}{4}a3(\lambda^{\phi 2}\;-\;1)(\lambda^{\theta 2}\;-\;1)}-3\} \end{array} \end{array}$$

and the Zhou-Fung strain energy function (Zhou and Fung, 1997):

(14) $$\begin{array}{c} \begin{array}{l} \psi =\frac{c}{2}\left\{\begin{array}{l} e^{\frac{1}{4}\;\left[a_{1}{\left(\lambda^{\phi 2}-1\right)}^{2}+a_{2}{\left(\lambda^{\theta 2}-1\right)}^{2}+2a_{3}\left(\lambda^{\phi 2}-1\right)\left(\lambda^{\theta 2}-1\right)\right]}\\ -\;\frac{1}{4}[a_{1}{\left(\lambda^{\phi 2}-1\right)}^{2}+a_{2}{\left(\lambda^{\theta 2}-1\right)}^{2}\\ +\;2a_{3}\left(\lambda^{\phi 2}-1\right)\left(\lambda^{\theta 2}-1\right)]-1 \end{array}\right\}\\ \;+\;\frac{1}{8}[b_{1}{\left(\lambda^{\phi 2}-1\right)}^{2}+b_{2}{\left(\lambda^{\theta 2}-1\right)}^{2}\\ \;+2b_{3}\left(\lambda^{\phi 2}-1\right)\left(\lambda^{\theta 2}-1\right)] \end{array} \end{array}$$

### Inverse estimate of the material parameters

The material parameters in Equations (10) or (12) or (13) or (14) are selected to minimize the objective function:

(15) $$f=\sum_{i\;=\;1}^{3}\sum_{j\;=\;1}^{N}\left[{\left(\sigma_{ij}^{\phi }-\sigma_{ij}^{\prime \phi }\right)}^{2}+{\left(\sigma_{ij}^{\theta }-\sigma_{ij}^{\prime \theta }\right)}^{2}\right]\;.$$

The minimization was performed using the Trust-Region-Reflective algorithm in MATLAB (More and Sorensen, 1983) which terminated when the objective function value is less than 10^ − 5^. In addition, the following RMS error (RMSE) is calculated to assess the curve-fitting quality:

(16) $$\varepsilon =\frac{\sqrt{\frac{1}{6N}\sum_{i\;=\;1}^{3}\sum_{j\;=\;1}^{N}\left[{\left(\sigma_{ij}^{\phi }-\sigma_{ij}^{\prime \phi }\right)}^{2}+{\left(\sigma_{ij}^{\theta }-\sigma_{ij}^{\prime \theta }\right)}^{2}\right]}}{\frac{1}{6N}\sum_{i\;=\;1}^{3}\sum_{j\;=\;1}^{N}\left[\sigma_{ij}^{\phi }+\sigma_{ij}^{\theta }\right]}\times 100\%.$$

It should be pointed out that the optimization process was conducted at points 1, 2, and 3 simultaneously rather than separately at each point. To secure a global minimum, the initial guesses of the parameters were chosen randomly within a suitable range, such as [0.01, 10] for *c*^1^, $\kappa_{1}^{1}$, $\kappa_{2}^{1}$, $\kappa_{4}^{1}$, *c*^2^, $\kappa_{1}^{2}$, $\kappa_{2}^{2}$, $\kappa_{4}^{2}$, *c*^3^, and $\kappa_{4}^{3}$, but [0.01, 3] for $\kappa_{3}^{1}$, $\kappa_{3}^{2}$, and $\kappa_{3}^{3}$. In those ranges, the optimized material parameters did not occur at the boundaries, and the curve fitting error was in the minimum. 80 initial guesses were generated randomly, followed by 80 optimization processes. The mean property constants and curve fitting errors were chosen to be the results. The detail of initial guesses on property constants optimization is given in Section Effects of Material Heterogeneity.

### The variable wall thickness

**Figure 4:**
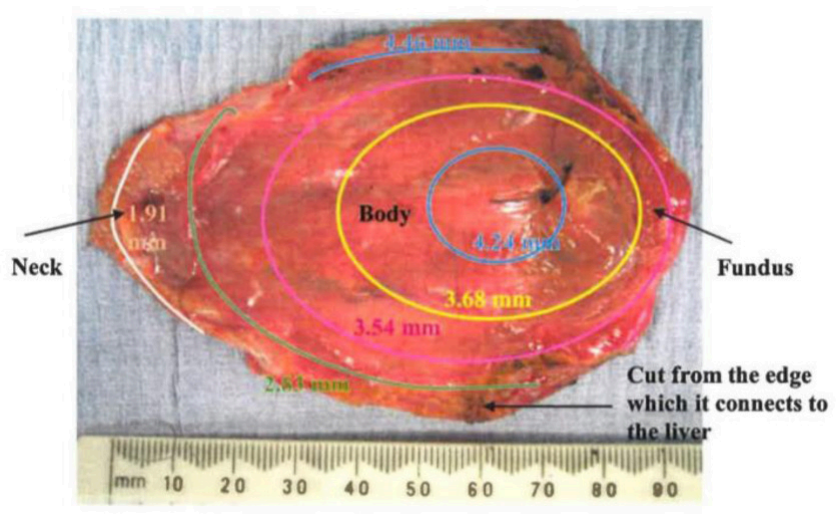
**A GB wall thickness profile, showing the thickest wall at the GB apex and thinnest wall near the neck, adopted from Su (2005) with permission**.

The GB wall thickness is related to the stress magnitude determined from the experimental images, as shown in Equations (7) and (8). This means that even when the pressure is the same, stresses can be different due to a varied thickness. This leads to different material parameters in the strain energy function in Equation (10). We now address the issue of the variable wall thickness of GB. A three-dimensional *in vivo* measurement of wall thickness of the GB was not yet available (Engel et al., 1980; Sanders, 1980; Prasad et al., 2008; Mohammed et al., 2010; Ugwu and Agwu, 2010); however, varying thickness of GB wall was measured *in vitro* with a digital slide caliper (Su, 2005; Khan et al., 2012). A contour of GB wall thickness is illustrated in Figure 4 (Su, 2005). It is observed that the thickness of the GB apex in the fundus increases to around 5 mm maximum and the wall of the neck is as thin as 2 mm. The ratio of the maximum thickness over the thickness of the body is 1.2.

In Khan et al. (2012), 62 GB samples were divided into three age groups; (10–20) years, (21–40) years, and (41–70) years. The thicknesses of these GBs were measured manually at the fundus, body and neck. It was identified that the maximum thickness was found on the neck, and the thinnest wall is located at the fundus. For the (41–70) years group, which coincides with the patient's age group for GB surgery in the paper, the ratio of the thickness at the fundus over the thickness of the body is 0.9. This is contrary to the finding in Su (2005). These ratios are used to examine the effect of a varying thickness.

### GB samples

The input data for our model were the geometrical parameters based on ultrasound images, and internal pressures at the start and end of emptying/refilling phase of ten GB samples from a previous study (Li et al., 2013). These geometrical parameters and internal pressures are shown in Table 1. Additionally, the geometrical parameters and pressure profile at 15 or more moments between the start and the end of refilling phase were interpolated according to the method in Li et al. (2011) and Equation (9). A uniform wall thickness was assumed at the start of refilling phase, i.e., *h*_11_ = *h*_21_ = *h*_31_ = 2.5 mm (Li et al., 2011, 2012, 2013). The stretch components of GB No. 1 over time are shown in Figure 2B. Note that the stretch-volume profiles are patient-specific.

**Table 1:** **Parameters of ten human GB samples, these parameters are for one dataset rather than an average of the whole set**.

**Moment**	**Parameter**	**GB sample No**.									
		**1**	**3**	**4**	**17**	**19**	**21**	**29**	**37**	**39**	**43**
At end of refilling	*p*_*N*_ (Pa)	1,466.5									
	*D*_1*N*_ (mm)	23.4	26.8	32.9	27.2	34.7	28.2	28.1	30.2	33.2	37.6
	*D*_2*N*_ (mm)	25.0	27.9	35.2	27.2	35.7	30.1	28.9	30.8	33.5	38.0
	*D*_3*N*_ (mm)	54.1	70.7	57.5	55.9	92.3	74.5	56.1	53.8	53.9	82.1
At start of refilling	*p*_1_ (Pa)	466.6									
	*D*_11_ (mm)	16.8	21.0	24.8	21.4	26.8	20.8	20.1	24.7	24.2	28.1
	*D*_21_ (mm)	18.2	21.2	25.8	20.7	29.2	24.2	22.7	24.7	26.1	29.7
	*D*_31_ (mm)	51.7	59.3	54.9	46.7	72.9	62.9	49.9	41.2	47.5	70.3
Ejection Fraction (EF) in 30 min (%)		4.5	11.4	13.3	32.4	49.4	66.3	37.8	77.0	60.1	2.7

*A uniform thickness, i.e., h_11_ = h_21_ = h_31_ = 2.5 mm, is assumed*.

In Table 1, the ejection fraction (EF) of a GB is defined as the ratio of the difference between the initial and emptied GB volumes at 30 min after venous injection of stimulator-CCK. Ethical approval for the use of data in Figure 4 and (Li et al., 2013) were approved by the ethical committees in the hospital where the studies were conducted, and the subjects gave informed consent to these studies.

### The solution of inverse problem

From Table 1, we had the ellipsoid model geometrical parameters at the beginning and the end of the refilling phase, which were the same as the end (30 min after CCK) and beginning of the emptying phase from the routine ultrasound images taken in hospital, as shown in Figure 1. As the least squares method required more scattered points than the number of parameters to be estimated, the ellipsoid model (Li et al., 2011) was interpolated over 15 or more time points for the emptying phase. The internal bile pressure is then given by Equation (9). These data were used to obtain the initial guess for the optimization process. The stretch and stress components were then computed, and the objective function and the RMSE were evaluated and compared to a given criterion of 10^ − 6^. If the criterion was not satisfied, a new guess based on the Trust-Region-Reflective algorithm would be generated, and the procedure repeated until the convergent result is reached.

## Results

### Effects of the initial guesses on material property constants

GB No.1 shown in Table 1 was randomly chosen to identify effects of initial guesses on the repeatability of inversely determined GB wall biomechanical property constants at points 1, 2, and 3. The initial guesses of the constants were generated randomly in the ranges for search of property constants mentioned in Section Inverse Estimate of the Material Parameters by normal distribution function in MATLAB and the numbers of initial guesses were specified 10, 20, 30, …, 130, respectively. The means of the determined property constants and RMSE as well as their standard error at 95% confidence level are illustrated in Table 2. The true value of these property constants should be equal to the mean ± its standard error at 95% confidence level.

**Table 2:** **Mean material property constants and standard error at 95% confidence level of GB No.1 with 2.5 mm uniform initial thickness at various initial guests**.

**No of initial guesses**	***c*^1^ (kPa)**	**$\kappa_{1}^{1}$ (kPa)**	**$\kappa_{2}^{1}$ (–)**	**$\kappa_{3}^{1}$ (kPa)**	**$\kappa_{4}^{1}$ (–)**	***c*^2^ (kPa)**	**$\kappa_{1}^{2}$ (kPa)**	**$\kappa_{2}^{2}$ (–)**	**$\kappa_{3}^{2}$ (kPa)**	**$\kappa_{4}^{2}$ (–)**	***c*^3^ (kPa)**	**$\kappa_{3}^{3}$ (kPa)**	**$\kappa_{4}^{3}$ (–)**	**ε (%)**	**Time costed (min)**
10	0.2509 ± 0.0340	2.1467 ± 0.0531	0.3263 ± 0.0170	0.5875 ± 0.0518	0.6255 ± 0.0782	0.0155 ± 0.0036	2.4547 ± 0.0058	0.2858 ± 0.0017	0.1048 ± 0.0075	1.5144 ± 0.0937	1.4855 ± 0.0088	2.4611 ± 0.0476	4.4470 ± 1.4226	7.2492 ± 0.0487	4.2
20	0.2169 ± 0.0222	2.1999 ± 0.0352	0.3105 ± 0.0109	0.6411 ± 0.0340	0.5532 ± 0.0419	0.0156 ± 0.0022	2.4547 ± 0.0036	0.2858 ± 0.0010	0.1042 ± 0.0047	1.5207 ± 0.0571	1.4808 ± 0.0080	2.4712 ± 0.0336	5.2825 ± 0.9006	7.1797 ± 0.0601	8.5
30	0.2136 ± 0.0239	2.2064 ± 0.0372	0.3089 ± 0.0112	0.6467 ± 0.0376	0.5531 ± 0.0478	0.0172 ± 0.0039	2.4519 ± 0.0064	0.2867 ± 0.0019	0.1030 ± 0.0066	1.5481 ± 0.0933	1.4798 ± 0.0058	2.4768 ± 0.0213	5.2257 ± 0.6993	7.1762 ± 0.0440	13.0
40	0.2234 ± 0.0219	2.1906 ± 0.0344	0.3136 ± 0.0105	0.6313 ± 0.0340	0.5734 ± 0.0429	0.0172 ± 0.0034	2.4520 ± 0.0055	0.2866 ± 0.0016	0.1031 ± 0.0055	1.5465 ± 0.0798	1.4802 ± 0.0067	2.4812 ± 0.0294	4.7908 ± 0.6233	7.1914 ± 0.0513	17.2
50	0.2268 ± 0.0185	2.1847 ± 0.0289	0.3154 ± 0.0090	0.6260 ± 0.0284	0.5794 ± 0.0395	0.0176 ± 0.0028	2.4512 ± 0.0045	0.2868 ± 0.0013	0.1018 ± 0.0048	1.5598 ± 0.0658	1.4831 ± 0.0046	2.4671 ± 0.0194	4.8035 ± 0.5842	7.2110 ± 0.0376	21.1
60	0.2269 ± 0.0146	2.1856 ± 0.0227	0.3147 ± 0.0069	0.6253 ± 0.0227	0.5765 ± 0.0299	0.0162 ± 0.0022	2.4537 ± 0.0036	0.2861 ± 0.0011	0.1042 ± 0.0038	1.5275 ± 0.0525	1.4826 ± 0.0050	2.4617 ± 0.0223	5.3255 ± 0.5797	7.2025 ± 0.0368	25.2
70	0.2231 ± 0.0117	2.1906 ± 0.0184	0.3131 ± 0.0057	0.6310 ± 0.0181	0.5667 ± 0.0242	0.0161 ± 0.0018	2.4536 ± 0.0030	0.2861 ± 0.0009	0.1043 ± 0.0034	1.5248 ± 0.0453	1.4842 ± 0.0042	2.4603 ± 0.0190	4.9036 ± 0.4887	7.2082 ± 0.0310	28.8
80	0.2180 ± 0.0131	2.1992 ± 0.0206	0.3105 ± 0.0064	0.6391 ± 0.0203	0.5603 ± 0.0267	0.0160 ± 0.0016	2.4538 ± 0.0025	0.2861 ± 0.0008	0.1041 ± 0.0030	1.5253 ± 0.0385	1.4801 ± 0.0040	2.4807 ± 0.0176	4.8695 ± 0.4743	7.1827 ± 0.0310	32.7
90	0.2182 ± 0.0129	2.1981 ± 0.0203	0.3113 ± 0.0062	0.6397 ± 0.0201	0.5592 ± 0.0249	0.0153 ± 0.0013	2.4550 ± 0.0022	0.2857 ± 0.0007	0.1056 ± 0.0025	1.5064 ± 0.0338	1.4815 ± 0.0044	2.4764 ± 0.0200	4.6928 ± 0.4266	7.1907 ± 0.0318	37.1
100	0.2114 ± 0.0124	2.2094 ± 0.0194	0.3075 ± 0.0060	0.6493 ± 0.0192	0.5494 ± 0.0248	0.0159 ± 0.0018	2.4541 ± 0.0030	0.2860 ± 0.0009	0.1049 ± 0.0030	1.5217 ± 0.0440	1.4815 ± 0.0041	2.4714 ± 0.0175	5.0405 ± 0.4098	7.1845 ± 0.0317	40.8
110	0.2182 ± 0.0134	2.1982 ± 0.0209	0.3115 ± 0.0064	0.6397 ± 0.0207	0.5646 ± 0.0283	0.0175 ± 0.0024	2.4515 ± 0.0039	0.2867 ± 0.0012	0.1029 ± 0.0035	1.5565 ± 0.0580	1.4799 ± 0.0033	2.4794 ± 0.0154	5.0577 ± 0.4053	7.1850 ± 0.0269	46.0
120	0.2184 ± 0.0101	2.1977 ± 0.0158	0.3112 ± 0.0048	0.6391 ± 0.0157	0.5578 ± 0.0194	0.0168 ± 0.0020	2.4526 ± 0.0033	0.2864 ± 0.0010	0.1033 ± 0.0029	1.5477 ± 0.0508	1.4797 ± 0.0032	2.4829 ± 0.0141	4.8604 ± 0.3984	7.1803 ± 0.0233	48.5
130	0.2153 ± 0.0105	2.2033 ± 0.0164	0.3094 ± 0.0051	0.6432 ± 0.0162	0.5564 ± 0.0228	0.0170 ± 0.0019	2.4523 ± 0.0030	0.2865 ± 0.0009	0.1033 ± 0.0029	1.5451 ± 0.0457	1.4815 ± 0.0039	2.4729 ± 0.0177	4.9616 ± 0.3444	7.1894 ± 0.0284	51.1

Note that the parameters at points 1–3 determined by the least squares method based on the Trust-Region-Reflective algorithm could not be repeated from one initial guess to another due to the complexity of the inverse problem. Considering the property constants determined from statistics point of view, however, the material parameters and their standard error at 95% confidence level inversely determined remained unchanged basically, especially when the number of initial guesses was 80 or more. This suggests that the biomechanical property constant values are repeatable in a statistical sense and are globally optimum. In the following sections, the property constants are extracted with 80 initial guesses at a computational cost of around 30 min. This time consumption is much less than 3.5–7.0 h based on the approach of ABAQUS 3D FEA plus MATLAB optimization solver in Li et al. (2013).

### Effects of material heterogeneity

The material parameters of heterogeneity inversely determined are listed in Table 3 and compared with those from the corresponding homogenous model in Li et al. (2013). The error ε in the homogenous model reflects the error in GB volume between image observation and homogenous model prediction.

**Table 3:** **Heterogeneous material parameters of ten GB samples determined inversely with model Equation (10) and compared with homogeneous model (Li et al., 2013) in uniform thickness ***h***_**11**_ = ***h***_**21**_ = ***h***_**31**_ = 2.5 mm**.

**GB No**.	**Model**	**Point *i***	***c*^*i*^ (kPa)**	**$\kappa_{1}^{i}$ (kPa)**	**$\kappa_{2}^{i}$ (–)**	**$\kappa_{3}^{i}$ (kPa)**	**$\kappa_{4}^{i}$ (–)**	**ε (%)**
1	Heterogeneous	1	0.2180 ± 0.0131	2.1992 ± 0.0206	0.3105 ± 0.0064	0.6391 ± 0.0203	0.5603 ± 0.0267	7.1827 ± 0.0310
		2	0.0160 ± 0.0016	2.4538 ± 0.0025	0.2861 ± 0.0008	0.1041 ± 0.0030	1.5253 ± 0.0385	
		3	1.4801 ± 0.0040	–	–	2.4807 ± 0.0176	4.8695 ± 0.4743	
	Homogeneous	1, 2, 3	2.3349	0.5977	0.8512	1.3952	1.0430	2.2
3	Heterogeneous	1	0.1742 ± 0.0076	3.6549 ± 0.0142	0.7242 ± 0.0053	0.8486 ± 0.0141	1.4082 ± 0.0281	1.9123 ± 0.0152
		2	0.0885 ± 0.0084	3.3645 ± 0.0150	0.4526 ± 0.0047	0.1987 ± 0.0144	0.9714 ± 0.1228	
		3	0.3487 ± 0.0001	–	–	0.9275 ± 0.0012	4.2149 ± 0.0085	
	Homogeneous	1, 2, 3	1.8375	4.7385	1.3538	0.8694	0.9568	3.9
4	Heterogeneous	1	0.2229 ± 0.0086	4.0107 ± 0.0159	0.4920 ± 0.0045	1.1588 ± 0.0160	0.8603 ± 0.0185	3.2065 ± 0.0092
		2	0.1669 ± 0.0091	3.9481 ± 0.0168	0.4622 ± 0.0047	0.5402 ± 0.0162	1.2017 ± 0.0454	
		3	0.9313 ± 0.0004	–	–	0.6766 ± 0.0043	6.2768 ± 0.0462	
	Homogeneous	1, 2, 3	2.1817	2.9539	0.7230	0.6578	1.0458	3.0
17	Heterogeneous	1	0.1484 ± 0.0080	3.3757 ± 0.0151	0.8418 ± 0.0065	0.6389 ± 0.0144	1.8043 ± 0.0455	5.1699 ± 0.0131
		2	0.2152 ± 0.0116	2.9225 ± 0.0205	0.5591 ± 0.0074	0.5387 ± 0.0204	0.9941 ± 0.0516	
		3	0.8548 ± 0.0004	–	–	0.1702 ± 0.0017	5.1634 ± 0.1110	
	Homogeneous	1, 2, 3	1.6810	2.9213	0.1161	0.4784	1.5530	2.5
19	Heterogeneous	1	0.5583 ± 0.0195	4.4503 ± 0.0362	0.4628 ± 0.0094	0.6047 ± 0.0349	1.0724 ± 0.1010	3.6488 ± 0.0141
		2	0.0978 ± 0.0078	6.7567 ± 0.0169	0.6573 ± 0.0049	0.0512 ± 0.0025	3.2080 ± 0.0823	
		3	0.5965 ± 0.0003	–	–	0.6651 ± 0.0042	1.1060 ± 0.0216	
	Homogeneous	1, 2, 3	2.2772	6.2427	0.1106	0.2182	0.8042	3.0
21	Heterogeneous	1	0.2321 ± 0.0088	6.1282 ± 0.0227	3.2180 ± 0.0189	1.7915 ± 0.0157	7.2733 ± 0.0529	7.8681 ± 0.0058
		2	0.0576 ± 0.0002	2.7506 ± 0.0005	0.0628 ± 0.0002	0.0101 ± 0.0001	0.1280 ± 0.0228	
		3	0.0188 ± 0.0005	–	–	1.8847 ± 0.0046	5.3105 ± 0.0167	
	Homogeneous	1, 2, 3	2.2309	3.0375	0.0176	0.2213	0.7755	3.4
29	Heterogeneous	1	0.3411 ± 0.0158	2.3990 ± 0.0247	0.2708 ± 0.0066	0.5445 ± 0.0249	0.4547 ± 0.0363	5.1186 ± 0.0165
		2	0.1083 ± 0.0081	3.5589 ± 0.0156	0.8243 ± 0.0065	0.4838 ± 0.0135	2.6977 ± 0.0584	
		3	1.2416 ± 0.0013	–	–	0.5071 ± 0.0059	3.1976 ± 0.2470	
	Homogeneous	1, 2, 3	2.0624	1.6658	0.7148	0.8237	1.1547	2.6
37	Heterogeneous	1	0.2382 ± 0.0126	4.1246 ± 0.0250	1.2410 ± 0.0120	1.0673 ± 0.0257	1.7882 ± 0.0579	6.3186 ± 0.0054
		2	0.2411 ± 0.0117	4.1189 ± 0.0233	1.2435 ± 0.0113	1.0612 ± 0.0236	1.7999 ± 0.0555	
		3	0.9575 ± 0.0002	–	–	0.0149 ± 0.0005	6.7694 ± 0.0517	
	Homogeneous	1, 2, 3	1.9243	4.3563	0.4350	0.1451	1.3890	2.5
39	Heterogeneous	1	0.3117 ± 0.0121	2.9031 ± 0.0199	0.2749 ± 0.0051	0.8384 ± 0.0197	0.5063 ± 0.0218	5.5799 ± 0.0092
		2	0.2700 ± 0.0127	3.6645 ± 0.0238	0.6873 ± 0.0084	0.8535 ± 0.0221	1.8349 ± 0.0444	
		3	1.8285 ± 0.0019	–	–	0.1972 ± 0.0062	3.0987 ± 0.2607	
	Homogeneous	1, 2, 3	2.4066	1.7295	0.5803	0.8437	1.1167	2.5

For all the GBs, the material parameter associated with the matrix in the heterogeneous model is around 10 times that of the homogenous model. For GB 3, 4, 17, 29, 37, 39, and 43, the mean values of fibers-related material parameters at points 1 and 2, $\kappa_{1}^{2}$ and $\kappa_{3}^{2}$, basically agree with κ_1_ and κ_3_ in the homogenous model, i.e., $\kappa_{1}^{2}$ ≈ (1–2) κ_1_ and $\kappa_{3}^{2}$ ≈ (1–2) κ_3_. For GB 1, 19, and 21, $\kappa_{1}^{2}$ and $\kappa_{3}^{2}$ are different from κ_1_ and κ_3_ in the homogenous model. On one hand, the material parameters in the heterogeneous model at point 1 and point 2 are similar, implying the heterogeneity of the GB wall along the circumference is small. This is especially true for GB 37 which has *D*_1_ ≈ *D*_2_; the points 1 and 2 share the same parameters. On the other hand, the parameters at point 3 differ substantially from the other two, suggesting a strong heterogeneity from GB body to fundus.

The first principal stresses of all the GB samples are compared in Table 4 with those predicted by the homogenous model in Li et al. (2013). These stresses are extracted at point 1 since the length of an ellipsoid major axis is the shortest through that point, resulting in the highest stress level there based on Equations (7) and (8) in the ϕ direction. It is shown that the homogenous model underestimate the stresses in the wall of GB1, 3, 4, 29, 39, and 43, and overestimates them for the remaining GBs. As a result, the relative error in the first principal stresses varies in a range of −11.4%~ +10.8% in comparison with the stresses in the homogenous model.

**Table 4:** **The first principal stresses in 10 GB samples wall estimated by using the homogenous model in Li et al. (2013) and the heterogeneous model Equation (10) in the present paper**.

**Stress (kPa)**	**GB sample No**.									
	**1**	**3**	**4**	**17**	**19**	**21**	**29**	**37**	**39**	**43**
Homogenous (Li et al., 2013)	9.75	12.38	13.66	11.89	17.11	14.41	12.17	12.78	13.26	17.09
Heterogeneous	10.86	12.40	14.49	11.40	16.25	14.09	14.41	11.41	14.96	17.85
Pain due to CCK	No	No	No	Yes	Yes	No	No	No	Yes	Yes

### Comparison with other constitutive models

The inversely estimated parameters are shown in Tables 5–7 for the Fung, Choi-Vito, and Zhou-Fung strain energy functions, respectively. The parameters in the Zhou-Fung model were as many as 17 in total at the three points; thus the number of time instants was increased to 30 in the optimization procedure.

**Table 5:** **Material parameters of Fung strain energy function Equation (14) inversely determined with uniform thickness ***h***_**11**_ = ***h***_**21**_ = ***h***_**31**_ = 2.5 mm**.

**GB No**.	**Point *i***	***c*^*i*^ (kPa)**	**$a_{1}^{i}$**	**$a_{2}^{i}$**	**$a_{3}^{i}$**	**ε (%)**
1	1	5.9207 ± 0.0444	1.0182 ± 0.0060	0.0102 ± 0.0002	0.6373 ± 0.0006	7.4833 ± 0.0119
	2	6.2282 ± 0.0398	1.4220 ± 0.0106	0.0755 ± 0.0064	0.0600 ± 0.0062	
	3	5.6380 ± 0.0916	–	5.6863 ± 0.0900	–	
3	1	4.4149 ± 0.0299	2.4513 ± 0.0135	0.0225 ± 0.0020	0.4580 ± 0.0013	6.8817 ± 0.0102
	2	6.6937 ± 0.0337	1.9242 ± 0.0123	0.1144 ± 0.0087	0.0725 ± 0.0086	
	3	2.5090 ± 0.0910	–	3.2397 ± 0.1120	–	
4	1	5.4869 ± 0.0489	1.9630 ± 0.0145	0.0102 ± 0.00010	0.5777 ± 0.0003	13.6283 ± 0.0140
	2	6.8674 ± 0.0568	2.0333 ± 0.0252	0.2729 ± 0.0233	0.2018 ± 0.0237	
	3	3.1520 ± 0.0934	–	3.5454 ± 0.0952	–	
17	1	3.6600 ± 0.0395	2.865 ± 0.0244	0.0419 ± 0.0034	0.3753 ± 0.0020	17.5786 ± 0.0106
	2	6.0800 ± 0.0578	1.8191 ± 0.0262	0.3547 ± 0.0227	0.1892 ± 0.0227	
	3	2.4674 ± 0.1469	–	2.7210 ± 0.1183	–	
19	1	7.6011 ± 0.0673	1.9387 ± 0.0144	0.0103 ± 0.0004	0.3693 ± 0.0002	12.3314 ± 0.0231
	2	6.6126 ± 0.0695	3.5333 ± 0.0413	0.2148 ± 0.0265	0.3362 ± 0.0264	
	3	3.2850 ± 0.1789	–	1.9863 ± 0.1098	–	
21	1	3.3914 ± 0.0674	4.8971 ± 0.0613	0.8129 ± 0.0153	0.5357 ± 0.0100	11.7094 ± 0.0132
	2	9.3404 ± 0.0119	0.9968 ± 0.0010	0.0100 ± 0.0001	0.0934 ± 0.0001	
	3	1.5754 ± 0.0672	–	7.3935 ± 0.1600	–	
29	1	6.324 ± 0.0650	1.1187 ± 0.0095	0.0100 ± 0.0001	0.5099 ± 0.0004	16.0779 ± 0.0202
	2	4.2754 ± 0.0757	2.7660 ± 0.0531	0.3330 ± 0.0414	0.5028 ± 0.0414	
	3	3.4407 ± 0.0716	–	3.5408 ± 0.0713	–	
37	1	3.5278 ± 0.0919	3.5628 ± 0.0604	0.1031 ± 0.0094	0.4602 ± 0.0055	24.8315 ± 0.0129
	2	3.4863 ± 0.0700	3.7051 ± 0.0844	0.7644 ± 0.0690	0.7056 ± 0.0684	
	3	2.6134 ± 0.2148	–	1.9942 ± 0.1109	–	
39	1	5.9501 ± 0.0708	1.2697 ± 0.0124	0.0100 ± 0.0001	0.6480 ± 0.0005	20.5731 ± 0.0203
	2	5.5450 ± 0.0627	2.1687 ± 0.0566	0.5208 ± 0.0539	0.4967 ± 0.0539	
	3	3.7173 ± 0.0586	–	3.9577 ± 0.0636	–	
43	1	7.7685 ± 0.0518	1.6277 ± 0.0086	0.0100 ± 0.0001	0.3810 ± 0.0002	17.0028 ± 0.0199
	2	7.6550 ± 0.0566	2.2885 ± 0.0319	0.3060 ± 0.0297	0.3350 ± 0.0300	
	3	3.1778 ± 0.0862	–	3.1777 ± 0.0862	–	

**Table 6:** **Material parameters of Choi-Vito strain energy function Equation (13) inversely determined with uniform thickness ***h***_**11**_ = ***h***_**21**_ = ***h***_**31**_ = 2.5 mm**.

**GB No**.	**Point *i***	***c*^*i*^ (kPa)**	**$a_{1}^{i}$**	**$a_{2}^{i}$**	**$a_{3}^{i}$**	**ε (%)**
1	1	6.5709 ± 0.0467	1.0196 ± 0.0103	0.1209 ± 0.0070	0.9865 ± 0.0068	7.2885 ± 0.0086
	2	6.2533 ± 0.0550	1.3934 ± 0.0102	0.0112 ± 0.0003	0.3090 ± 0.0030	
	3	5.6432 ± 0.1665	–	5.7457 ± 0.1631	–	
3	1	6.7592 ± 0.0606	2.8159 ± 0.0111	0.6914 ± 0.0309	1.0173 ± 0.0838	6.8731 ± 0.0170
	2	6.1800 ± 0.0362	2.0092 ± 0.0094	0.0245 ± 0.0023	0.4406 ± 0.0054	
	3	2.4858 ± 0.1920	–	3.4989 ± 0.2282	–	
4	1	6.3626 ± 0.0556	2.0862 ± 0.0127	0.4669 ± 0.0211	1.2218 ± 0.04994	13.2913 ± 0.0049
	2	6.0338 ± 0.0369	2.1638 ± 0.0116	0.0814 ± 0.0056	1.1084 ± 0.0120	
	3	3.2084 ± 0.2091	–	3.6669 ± 0.19996	–	
17	1	3.5904 ± 0.0691	3.5174 ± 0.0342	0.8399 ± 0.0354	0.9713 ± 0.1038	17.4151 ± 0.0056
	2	4.4094 ± 0.0362	2.2944 ± 0.0147	0.2778 ± 0.0170	1.2093 ± 0.0349	
	3	2.7237 ± 0.2346	–	2.7146 ± 0.2294	–	
19	1	8.3497 ± 0.0547	1.9024 ± 0.0123	0.1462 ± 0.0066	1.1344 ± 0.0123	11.8413 ± 0.0103
	2	6.3147 ± 0.0600	3.6558 ± 0.0276	0.0840 ± 0.0110	1.1832 ± 0.0276	
	3	4.0103 ± 0.1681	–	1.6285 ± 0.0616	–	
21	1	3.0469 ± 0.0164	8.9908 ± 0.0453	4.3924 ± 0.0317	0.6240 ± 0.0482	12.1795 ± 0.0184
	2	9.2992 ± 0.0231	1.0055 ± 0.0020	0.0026 ± 0.0001	0.0277 ± 0.0002	
	3	1.4123 ± 0.0302	–	8.0059 ± 0.1113	–	
29	1	6.8779 ± 0.0807	1.1716 ± 0.0138	0.1738 ± 0.0076	0.8076 ± 0.0109	15.8654 ± 0.0173
	2	3.1266 ± 0.0826	3.7813 ± 0.0497	0.4507 ± 0.0607	1.8447 ± 0.1480	
	3	3.5246 ± 0.1540	–	3.5659 ± 0.1611	–	
37	1	3.4254 ± 0.0991	4.8591 ± 0.1016	1.5987 ± 0.0496	0.9439 ± 0.1021	24.6237 ± 0.0101
	2	2.7583 ± 0.1002	5.3099 ± 0.1160	1.2692 ± 0.0877	2.5030 ± 0.2218	
	3	2.5060 ± 0.2000	–	2.0923 ± 0.1252	–	
39	1	6.9593 ± 0.0467	1.3088 ± 0.0127	0.2592 ± 0.0121	1.0019 ± 0.0184	20.2444 ± 0.0071
	2	3.9188 ± 0.0587	2.9796 ± 0.0358	0.6190 ± 0.0436	2.1451 ± 0.0932	
	3	3.6297 ± 0.1896	–	4.2443 ± 0.2282	–	
43	1	8.5402 ± 0.0387	1.5659 ± 0.0074	0.0782 ± 0.0040	1.0716 ± 0.0060	16.2154 ± 0.0084
	2	6.9974 ± 0.0440	2.3804 ± 0.0143	0.0865 ± 0.0070	1.4685 ± 0.0132	
	3	3.5295 ± 0.2111	–	3.2640 ± 0.1878	–	

**Table 7:** **Material parameters of Zhou-Fung strain energy function Equation (14) inversely determined with uniform thickness ***h***_**11**_ = ***h***_**21**_ = ***h***_**31**_ = 2.5 mm**.

**GB No**.	**Point *i***	***c*^*i*^ (kPa)**	**$a_{1}^{i}$**	**$a_{2}^{i}$**	**$a_{3}^{i}$**	**$b_{1}^{i}$**	**$b_{2}^{i}$**	**$b_{3}^{i}$**	**ε (%)**
1	1	0.8073 ± 0.1082	2.0643 ± 0.1619	0.6341 ± 0.0806	0.5654 ± 0.0920	8.0082 ± 0.1667	1.6571 ± 0.1635	2.3635 ± 0.1627	13.8872 ± 0.0363
	2	0.7743 ± 0.0974	2.5808 ± 0.1633	0.7208 ± 0.0566	0.5077 ± 0.0877	9.8112 ± 0.0320	0.2040 ± 0.0241	0.2899 ± 0.0234	
	3	8.7721 ± 0.2652	–	9.2796 ± 0.2362	–	–	9.9707 ± 0.0095	–	
3	1	1.6195 ± 0.1581	3.1320 ± 0.2319	0.9319 ± 0.1349	0.9728 ± 0.1115	12.8219 ± 0.1550	1.4955 ± 0.1487	3.3015 ± 0.1511	6.4490 ± 0.0178
	2	1.2704 ± 0.1266	3.6118 ± 0.1468	0.1869 ± 0.0356	0.2755 ± 0.0562	13.4187 ± 0.0686	0.4733 ± 0.0459	0.8521 ± 0.0497	
	3	4.4095 ± 0.1615	–	4.1456 ± 0.0669	–	–	6.3141 ± 0.0108	–	
4	1	1.1620 ± 0.1257	3.2066 ± 0.3284	0.9681 ± 0.0987	0.7732 ± 0.0997	13.5437 ± 0.2035	2.0458 ± 0.2009	4.3685 ± 0.2090	13.0342 ± 0.0101
	2	1.0549 ± 0.1577	3.7930 ± 0.3425	0.8367 ± 0.1157	0.7100 ± 0.0833	14.7885 ± 0.1161	0.9880 ± 0.1008	2.4757 ± 0.1257	
	3	5.3204 ± 0.2107	–	4.8034 ± 0.0878	–	–	8.4977 ± 0.0886	–	
17	1	1.2488 ± 0.1413	3.7198 ± 0.2387	1.0768 ± 0.1045	1.1118 ± 0.1301	12.3554 ± 0.1548	1.2096 ± 0.1320	2.5024 ± 0.1405	17.0760 ± 0.0062
	2	0.7638 ± 0.1137	3.8420 ± 0.2851	0.9075 ± 0.1263	0.8016 ± 0.1500	11.2526 ± 0.1456	1.2561 ± 0.1235	2.3117 ± 0.1159	
	3	4.3245 ± 0.2086	–	4.0995 ± 0.1127	–	–	4.5930 ± 0.0455	–	
19	1	0.7828 ± 0.1307	4.6629 ± 0.3631	0.4719 ± 0.1150	0.4482 ± 0.1186	17.2832 ± 0.1329	1.2589 ± 0.1232	4.9771 ± 0.1244	11.0323 ± 0.0067
	2	1.4217 ± 0.1348	4.2640 ± 0.3540	1.5276 ± 0.1483	1.3113 ± 0.1283	25.9704 ± 0.1174	0.9578 ± 0.1076	2.0420 ± 0.1098	
	3	2.2564 ± 0.2448	–	1.3357 ± 0.1134	–	–	6.8376 ± 0.0216	–	
21	1	0.6073 ± 0.1682	5.5783 ± 0.7122	0.3904 ± 0.1108	0.3309 ± 0.0958	11.6934 ± 0.2064	2.6685 ± 0.2035	3.1046 ± 0.2028	5.5210 ± 0.0255
	2	1.1479 ± 0.1651	5.7567 ± 0.4519	1.3359 ± 0.1117	1.0352 ± 0.1403	18.4767 ± 0.0373	0.0749 ± 0.0118	0.1278 ± 0.0175	
	3	4.1435 ± 0.2240	–	3.3335 ± 0.0929	–	–	9.4489 ± 0.0439	–	
29	1	0.5637 ± 0.1207	2.9603 ± 0.3071	0.5336 ± 0.1119	0.4604 ± 0.0878	9.7022 ± 0.1465	1.8628 ± 0.1405	2.2909 ± 0.1386	15.0643 ± 0.0104
	2	1.0935 ± 0.1195	3.6787 ± 0.2249	1.6821 ± 0.1013	1.2663 ± 0.1381	13.5427 ± 0.1392	1.0954 ± 0.1270	1.7467 ± 0.1368	
	3	6.5934 ± 0.1729	–	6.5834 ± 0.1186	–	–	8.8734 ± 0.1268	–	
37	1	0.9666 ± 0.1439	3.1314 ± 0.1468	0.3768 ± 0.1183	0.4541 ± 0.1581	10.8332 ± 0.1713	2.1080 ± 0.1282	3.4023 ± 0.1355	22.8530 ± 0.0189
	2	4.3316 ± 0.1941	6.3448 ± 0.1610	1.5052 ± 0.1046	2.8789 ± 0.1302	18.7453 ± 0.1423	0.8814 ± 0.1296	5.9936 ± 0.1298	
	3	1.8696 ± 0.2859	–	1.0830 ± 0.1926	–	–	6.0539 ± 0.0198	–	
39	1	0.8969 ± 0.1399	1.8939 ± 0.1194	0.6789 ± 0.1009	0.6465 ± 0.1432	10.6963 ± 0.1726	2.1998 ± 0.1675	3.1981 ± 0.1706	19.7368 ± 0.0085
	2	1.3454 ± 0.1326	2.4035 ± 0.1253	1.5162 ± 0.0992	1.2377 ± 0.1315	13.2262 ± 0.2109	1.8452 ± 0.1971	3.6513 ± 0.1950	
	3	7.3471 ± 0.1801	–	7.3861 ± 0.0921	–	–	9.1861 ± 0.1025	–	
43	1	1.0615 ± 0.1756	2.5564 ± 0.1709	0.3917 ± 0.0843	0.4254 ± 0.1054	15.9433 ± 0.1504	1.5190 ± 0.1458	4.0383 ± 0.1455	15.3788 ± 0.0088
	2	1.3838 ± 0.1592	2.8815 ± 0.1915	1.2693 ± 0.0918	0.8113 ± 0.1182	18.9654 ± 0.0929	0.7135 ± 0.0829	4.3854 ± 0.0848	
	3	4.8098 ± 0.2783	–	4.4178 ± 0.1503	–	–	9.2673 ± 0.0868	–	

**Figure 5:**
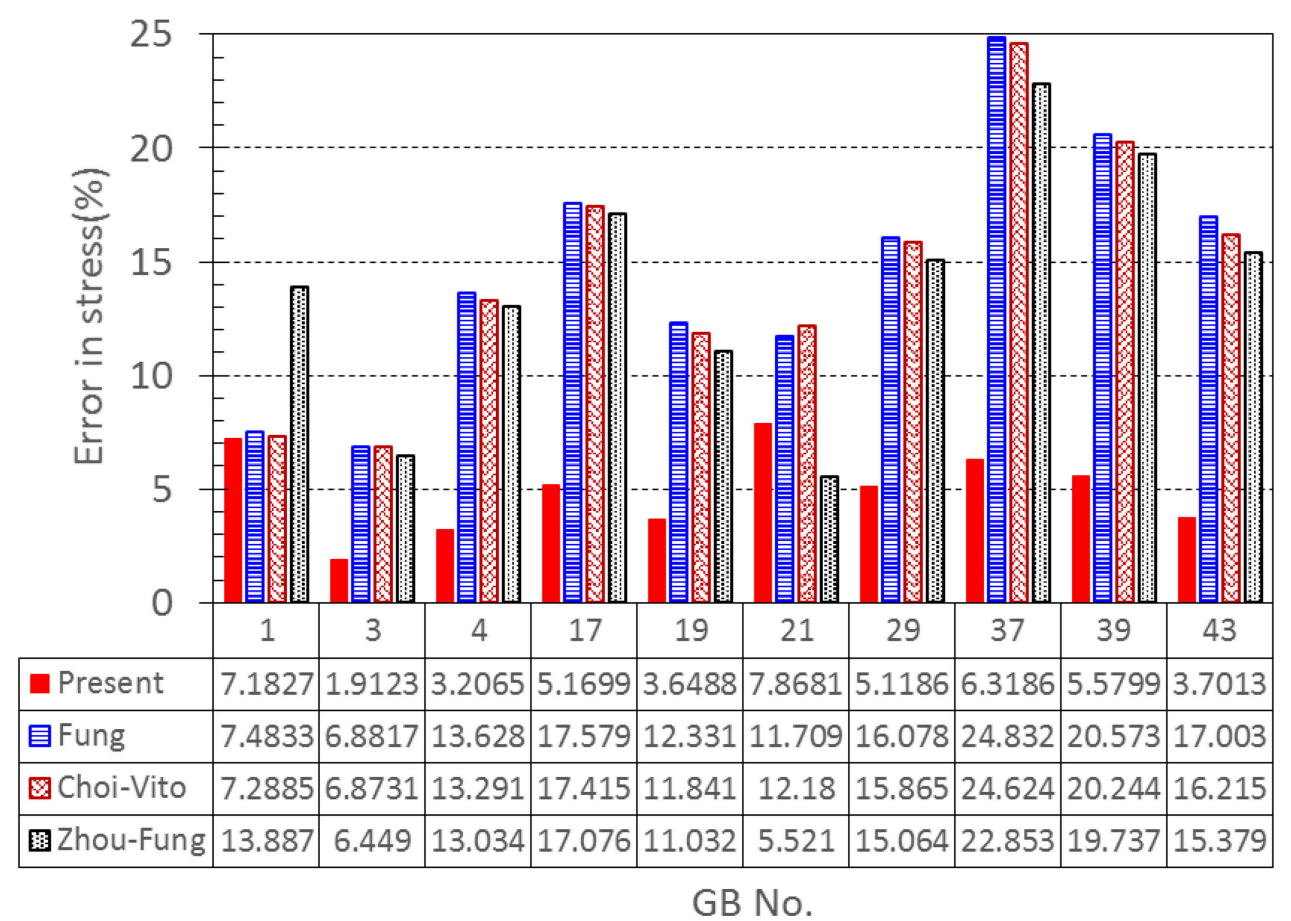
**A comparison of errors in the least-squares stress curve fitting between the present constitutive law and existing laws proposed by Fung, Choi-Vito, and Zhou-Fung, respectively**.

Our results show that even though the model parameters using these strain energy functions can also be inversely determined, the errors in stress are quite large. For instance, the mean errors are 14.8, 14.6, and 14.0% for the Fung, Choi-Vito and Zhou-Fung strain energy functions, respectively, while the structure-based model Equation (10) yields a mean error of 5.0% only (Figure 5).

### Variation of the GB wall thickness

**Figure 6:**
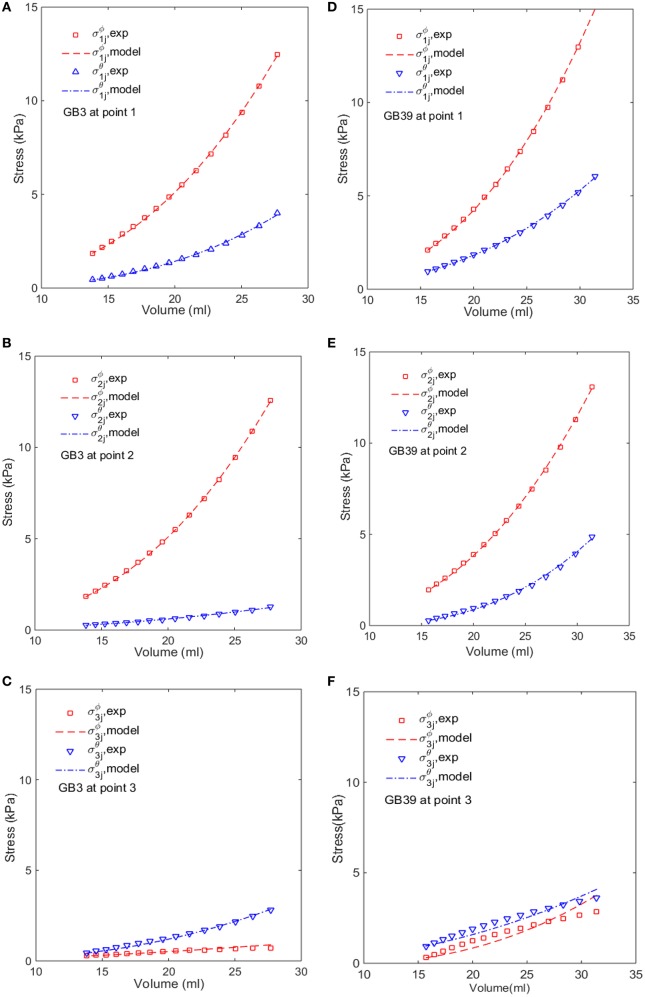
**Comparison of the modeled (lines) and estimated (symbols) circumferential and longitudinal stresses with the image-based ellipsoid membrane mechanic model at points 1, 2, 3, for GB 3 (A–C)**, and GB 39 **(D–F)**.

We notice from Table 3 that there are some large errors ranged between 5.2 and 7.8% for the parameters estimated for five GB samples: 1, 17, 21, 37, and 39. To identify the cause of the errors, the stress-volume curves of GB 3 and 39 at points 1, 2, and 3 are shown in Figure 6. The predicted stress agrees well with the observations at points 1 and 2, but not so well at point 3. In the following, we show that this is due to the uniform wall thickness assumption used in the model.

In Section Comparison with Other Constitutive Models, the GB wall heterogeneity mainly occurs in the apex region, resulting in poor agreement in the stress, as shown in Figure 6. Therefore, we altered the GB wall thickness at the apex to examine the effect of varying thickness. First, the apex thickness was changed to 3.0 mm, based on the ratio of 1.2 found in Su (2005), and kept at 2.5 mm at points 1 and 2. The extracted pointwise mechanical properties in Equation (10) are shown in Table 8.

**Table 8:** **Material parameters inversely determined with model Equation (10) and variable thicknesses: ***h***_**11**_ = ***h***_**21**_ = 2.5 mm, and ***h***_**31**_ = 3.0 mm**.

**GB No**.	**Point *i***	***c*^*i*^ (kPa)**	**$\kappa_{1}^{i}$ (kPa)**	**$\kappa_{2}^{i}$**	**$\kappa_{3}^{i}$ (kPa)**	**$\kappa_{4}^{i}$**	**ε (%)**
1	1	0.2146 ± 0.0118	2.2040 ± 0.0186	0.3090 ± 0.0056	0.6441 ± 0.0185	0.5514 ± 0.0218	5.9256 ± 0.0297
	2	0.0106 ± 0.0029	2.4626 ± 0.0047	0.2836 ± 0.0014	0.1089 ± 0.1089	1.5081 ± 0.0662	
	3	1.1918 ± 0.0046	–	–	2.2841 ± 0.0212	4.8171 ± 0.4371	
3	1	0.1739 ± 0.0077	3.6555 ± 0.0144	0.7239 ± 0.0053	0.8493 ± 0.0142	1.4068 ± 0.0291	1.8261 ± 0.0173
	2	0.0926 ± 0.0072	3.3569 ± 0.0130	0.4549 ± 0.0040	0.1907 ± 0.0123	0.9927 ± 0.1021	
	3	0.2897 ± 0.0002	–	–	0.7816 ± 0.0026	4.1440 ± 0.0214	
4	1	0.2299 ± 0.0087	3.9979 ± 0.0161	0.4956 ± 0.0046	1.1460 ± 0.0161	0.8751 ± 0.0196	2.8756 ± 0.0119
	2	0.1633 ± 0.0095	3.9544 ± 0.0174	0.4604 ± 0.0049	0.5469 ± 0.0169	1.1846 ± 0.0468	
	3	0.7727 ± 0.0003	–	–	0.6013 ± 0.0036	5.8020 ± 0.0544	
17	1	0.1572 ± 0.0086	3.3594 ± 0.0160	0.8488 ± 0.0070	0.6231 ± 0.0152	1.8528 ± 0.0515	4.6031 ± 0.0195
	2	0.2326 ± 0.0138	2.8920 ± 0.0241	0.5705 ± 0.0089	0.5087 ± 0.0238	1.0820 ± 0.0671	
	3	0.7162 ± 0.0007	–	–	0.1522 ± 0.0020	4.2194 ± 0.1741	
19	1	0.5454 ± 0.0152	4.4740 ± 0.0284	0.4564 ± 0.0073	0.6271 ± 0.0276	0.9859 ± 0.0699	3.2060 ± 0.0129
	2	0.0918 ± 0.0076	6.7692 ± 0.0165	0.6541 ± 0.0049	0.0493 ± 0.0025	3.1356 ± 0.0818	
	3	0.4974 ± 0.0003	–	–	0.5500 ± 0.0042	1.1336 ± 0.0261	
21	1	0.2266 ± 0.0094	6.1367 ± 0.0238	3.2163 ± 0.0194	1.8135 ± 0.0201	7.1830 ± 0.0671	7.5871 ± 0.0058
	2	0.0577 ± 0.0002	2.7500 ± 0.0006	0.0631 ± 0.0002	0.0101 ± 0.0002	0.1368 ± 0.0252	
	3	0.0157 ± 0.0005	–	–	1.5921 ± 0.0060	5.2078 ± 0.0272	
29	1	0.3272 ± 0.0156	2.4211 ± 0.0244	0.2648 ± 0.0065	0.5663 ± 0.0245	0.4261 ± 0.0358	4.4857 ± 0.0208
	2	0.1032 ± 0.0063	3.5691 ± 0.0120	0.8197 ± 0.0049	0.4906 ± 0.0105	2.6659 ± 0.0461	
	3	1.0348 ± 0.0013	–	–	0.4320 ± 0.0059	2.6961 ± 0.2625	
37	1	0.2469 ± 0.0126	4.1076 ± 0.0252	1.2490 ± 0.0122	1.0492 ± 0.0253	1.8288 ± 0.0601	5.5412 ± 0.0075
	2	0.2414 ± 0.0140	4.1183 ± 0.0280	1.2440 ± 0.0136	1.0606 ± 0.0283	1.8085 ± 0.0664	
	3	0.7965 ± 0.0002	–	–	0.0153 ± 0.0005	6.3465 ± 0.0628	
39	1	0.2999 ± 0.0131	2.9229 ± 0.0217	0.2699 ± 0.0054	0.8575 ± 0.0216	0.4888 ± 0.0222	4.8833 ± 0.0117
	2	0.2650 ± 0.0123	3.6739 ± 0.0230	0.6839 ± 0.0081	0.8623 ± 0.0215	1.8161 ± 0.0420	
	3	1.5221 ± 0.0023	–	–	0.1718 ± 0.0083	2.8898 ± 0.2615	
43	1	0.3508 ± 0.0156	4.3353 ± 0.0275	0.2502 ± 0.0054	0.7968 ± 0.0275	0.5590 ± 0.0371	3.2537 ± 0.0129
	2	0.2305 ± 0.0099	5.3712 ± 0.0192	0.4119 ± 0.0044	0.7883 ± 0.0181	1.5736 ± 0.0399	
	3	1.1491 ± 0.0005	–	–	0.2174 ± 0.0031	3.0536 ± 0.2349	

The relative changes in these 13 parameters are tabulated in Table 9. The increased thickness at the apex by 20% has a considerable effect on the material parameters, with changes up to 30%, in particular, on $\kappa_{4}^{1}$, *c*^2^, $\kappa_{4}^{2}$, *c*^3^, $\kappa_{3}^{3}$, and $\kappa_{4}^{3}$, which are associated with the properties of the matrix and the longitudinal fibers. This is very different to the membrane theory, in which the Young's modulus is independent of the membrane thickness (Timoshenko and Woinowsky-Krieger, 1959).

**Table 9:** **Relative changes in the parameters due to varied wall thickness**.

**GB No**.	**Point *i***	**Δ*c*^*i*^/*c*^*i*^ (%)**	**$\Delta \kappa_{1}^{i}/\kappa_{1}^{i}$ (%)**	**$\Delta \kappa_{2}^{i}/\kappa_{2}^{i}$ (%)**	**$\Delta \kappa_{3}^{i}/\kappa_{3}^{i}$ (%)**	**$\Delta \kappa_{4}^{i}/\kappa_{4}^{i}$ (%)**	***Δε* (%)**
1	1	−1.5229	0.2183	−0.4831	0.7824	−1.5884	−1.2571
	2	−33.7500	0.3586	−0.8738	4.61095	−1.12765	
	3	−19.4784	−	−	−7.9252	−1.0761	
3	1	−0.1722	0.01642	−0.0414	0.0825	−0.0994	−0.0862
	2	4.6328	−0.2259	0.5082	−4.0262	2.1927	
	3	−16.9200	−	−	−15.7305	−1.6821	
4	1	3.14042	−0.3192	0.7317	−1.1046	1.7203	−0.3309
	2	−2.1570	0.1596	−0.38944	1.2403	−1.4230	
	3	−17.0300	−	−	−11.1292	−7.5644	
17	1	5.9299	−0.4829	0.8316	−2.4730	2.6880	−0.5668
	2	8.0855	−1.0436	2.0390	−5.5690	8.8422	
	3	−16.2143	−	−	−10.5758	−18.2825	
19	1	−2.3106	0.5325	−1.3829	3.7043	−8.0660	−0.4428
	2	−6.1350	0.1850	−0.4868	−3.7109	−2.2569	
	3	−16.6136	−	−	−17.3057	2.4955	
21	1	−2.3697	0.1387	−0.0528	1.2280	−1.2415	−0.2810
	2	0.1736	−0.0218	0.47771	0	6.8750	
	3	−16.4894	−	−	−15.5250	−1.9339	
29	1	−4.0751	0.9212	−2.2157	4.0037	−6.2898	−0.6329
	2	−4.7091	0.2866	−0.5581	1.4055	−1.1788	
	3	−16.6559	−	−	−14.8097	−15.6836	
37	1	3.6524	−0.4122	0.6446	−1.6959	2.2704	−0.7774
	2	0.1244	−0.0146	0.0402	−0.0565	0.4778	
	3	−16.8146	−	−	2.6846	−6.2472	
39	1	−3.7857	0.6820	−1.8188	2.2781	−3.4565	−0.6966
	2	−1.8519	0.2565	−0.4947	1.0310	−1.0246	
	3	−16.7569	−	−	−12.8803	−6.7415	
43	1	−3.7322	0.56367	−1.8439	3.0656	−5.6381	−0.4476
	2	−3.2326	0.28192	−0.8426	1.8476	−1.9197	
	3	−16.5808	−	−	−13.4554	−17.0601	

*Δ*c*^*i*^, $\Delta \kappa_{1}^{i}$, $\Delta \kappa_{2}^{i}$, $\Delta \kappa_{3}^{i}$, $\Delta \kappa_{4}^{i}$ and Δε are the differences of these parameters and error between the case of h_31_ = 3.0 mm thick apex wall and the case of 2.5 mm uniform GB wall, *c*^i^, $\kappa_{1}^{i}$, $\kappa_{2}^{i}$, $\kappa_{3}^{i}$$\kappa_{4}^{i}$ are the parameters for the 2.5 mm uniform GB wall*.

**Figure 7:**
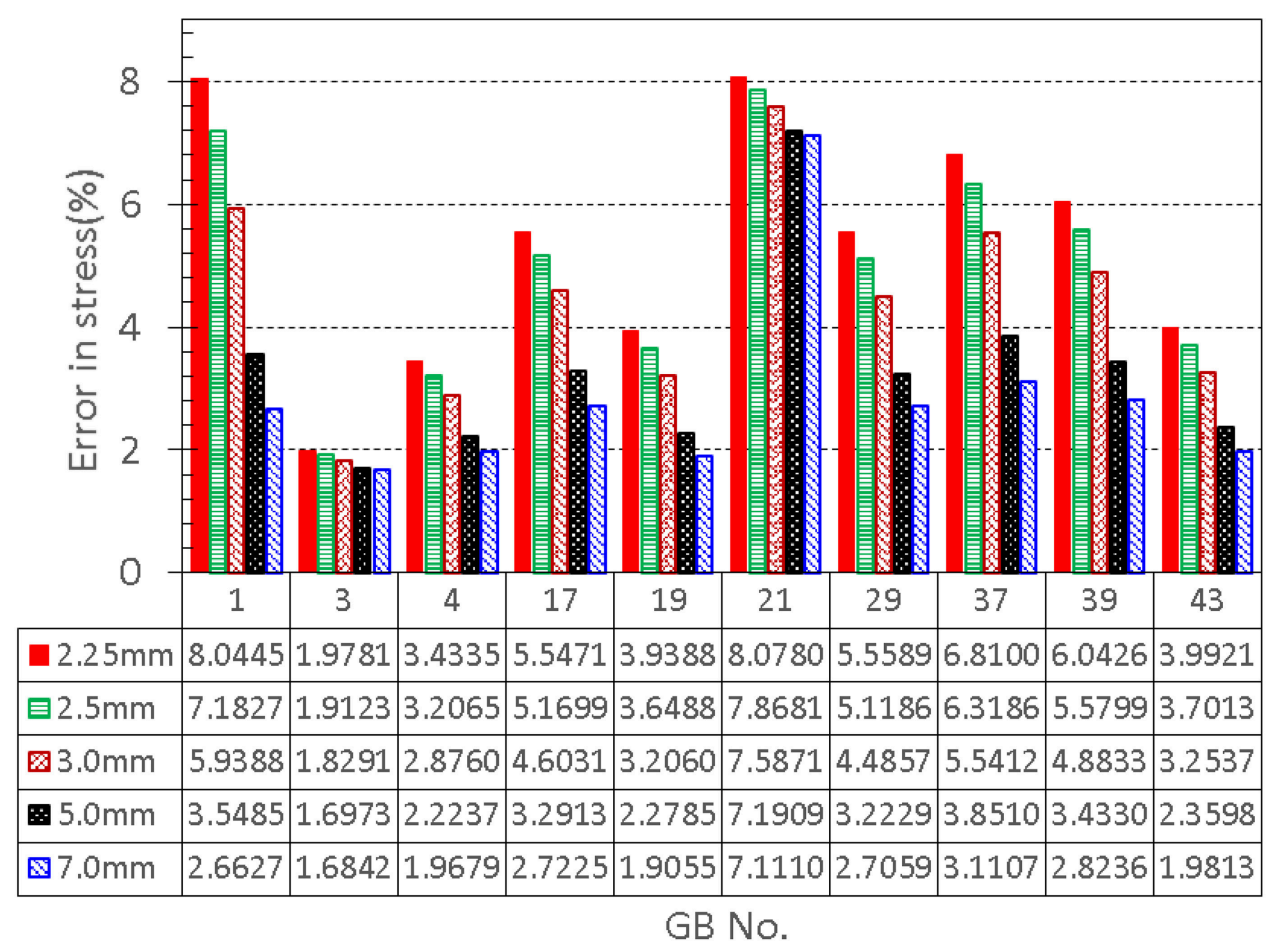
**Effect of GB wall thickness at the apex on the error in stress, the thickness at the apex is varied to be 2.25, 2.5, 3.0, 5.0, and 7.0 mm, respectively, while the thickness at the other two points 1 and 2 is kept to be 2.5 mm**.

We also found that an increased *h*_31_ could lower the error in the stress between the model production and the observation. If we increase *h*_31_ to 5.0 mm, the error reduces by 2.5%. Further increase in thickness does not decrease the error much, see Figure 7. Interestingly, 5.0 mm apex thickness seems to agree with measurement in Su (2005). Finally, if *h*_31_ is reduced to be 10% thinner than *h*_11_ and *h*_21_, 2.25 m, according to Khan et al. (2012), then the errors in the stresses are greater, as shown in Figure 7. Thus, the observation that apex thickness was thinner than the GB body in Khan et al. (2012) did not agree with the results from the cohort of GB samples used here.

Note that in Khan et al. (2012) post-mortem samples from “unclaimed bodies” were used and so would not have been fresh. When left *in situ* the bile will start to break down the gallbladder wall–a process known as autolysis. Therefore, the results for wall thickness might not be reliable. Samples in Su (2005), on the other hand were obtained fresh from the operating theater and washed immediately. So we would have more faith in the results in Su (2005).

### Comparison with animal test

**Figure 8:**
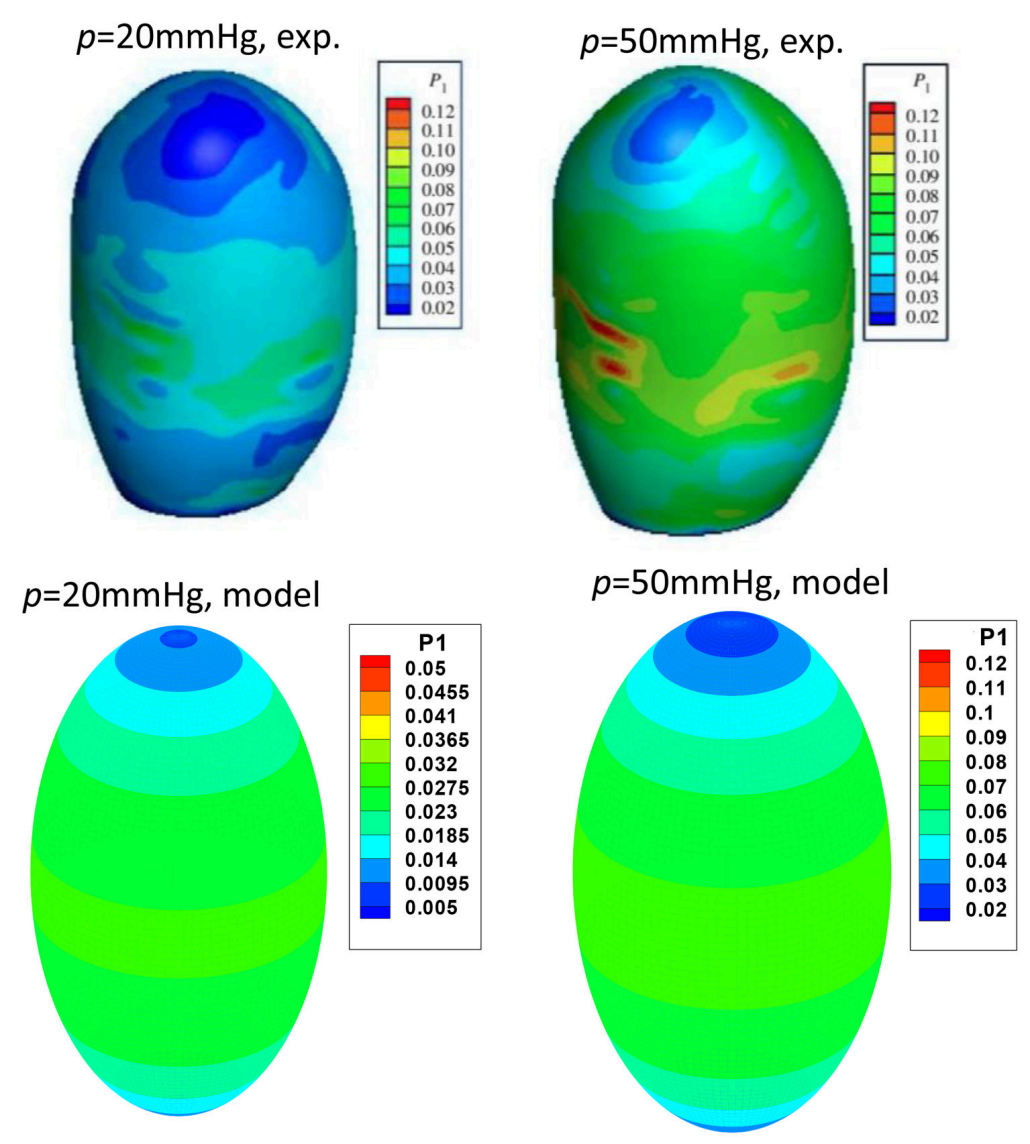
**Comparison of the peak tension from the ellipsoid model with the ***in vitro*** experimental tension of a lamb GB (Genovese et al., 2014) at the internal pressure of 20 and 50 mmHg, respectively, two plots in the top row are from Genovese et al. (2014), with permission**.

Our ellipsoid model is different from the patient specific GB geometries. One may ask if such a simple model is of any practical use. To answer this question, we compared our model prediction with the *in vitro* measurements of a lamb GB (Genovese et al., 2014). In Genovese et al. (2014), a lamb GB was harvested and inflated at a pressure up to 50 mmHg, then a series points on the GB outside surface were tracked optically, and the strain fields were estimated from fitting curves of these points. The tension/stress fields were then calculated by using the elastic membrane model and solved numerically. In our model, we only used the diameters from Genovese et al. (2014) as the two minor axis lengths *D*_1_ and *D*_2_, respectively. The stress field, hence tension, are obtained analytically from Equation (7). The results are shown in Figure 8, where the comparisons of the second Piola-Kirchhoff surface tensions are plotted for pressure *p* = 20 and 50 mmHg (we couldn't compare the results at *p* = 3.5 mmHg, as the tension profile in Genovese et al. (2014) seems to be unrealistically large, which is possibly due to a typo in the color map scale).

The overall agreement is encouraging; in both our model and experiments, the highest tension is found near the GB equator, and the minimum tension occurs at the apex. The values of the predicted surface tension are also in good agreement with the experimental data except some isolated tension spots due to the rapid change in the wall curvature of the lamb GB. The predicted surface tension magnitude near the GB body/equator is in a range of 0.023–0.027 N/mm, compared with 0.03–0.04 N/mm in the experiments at 20 mmHg pressure. Likewise, the predicted tension is in a range of 0.063–0.073 N/mm, compared to the range of 0.06–0.08 N/mm in the experiments at 50 mmHg.

## Discussion

Ultrasonography is a common method for monitoring GB volume variations in daily diagnosis (Dodds et al., 1985; Portincasa et al., 2003; Ugwu and Agwu, 2010). Although a detailed 3D model is more accurate, simplified geometry models are fast and therefore frequently used in clinical assessment. When an ellipsoidal model is used to estimate GB volume based on the images scanned during emptying phase, the error of the model in GB volume is about 0.8 ± 0.1 ml. This compares better to the error of 2.1 ± 0.2 ml if using sum-of-cylinder method (Dodds et al., 1985). To assess if the simplified model could predict the stress distribution of a realistic GB sample, we also compared our model prediction with the surface tension data for a lamb GB, and the overall agreement was surprisingly good.

We comment that the segmentation error of estimating the GB diameter from a GB image is usually around 4.31–7.21% (Bocchi and Rogai, 2011). To address the effect of this error on the GB wall material parameters, we introduce a random error (or noise) of 4.31–7.21% in the major axis lengths, i.e.:

(17) $$\varepsilon_{seg}=0.0431\;+\;rand\;\times \;\left(0.0721-0.0431\right)$$

where *rand* is the inner random function in MATLAB to generate a random number in value 0–1. Then we run the inverse heterogeneous problem code with these noisy data for a number of GB sample, say No. 1, 3, 17, and 21. The parameters estimated are compared in Table 10 against those without the noise.

**Table 10:** **Heterogeneous material parameters of four GB samples inversely determined with Equation (10) and uniform thickness ***h***_**11**_ = ***h***_**21**_ = ***h***_**31**_ = 2.5 mm when the error in segmentation is considered**.

**GB No**.	**Point *i***	**With segmentation error**	***c*^*i*^ (kPa)**	**$\kappa_{1}^{i}$ (kPa)**	**$\kappa_{2}^{i}$ (–)**	**$\kappa_{3}^{i}$ (kPa)**	**$\kappa_{4}^{i}$ (–)**
1	1	No	0.2180 ± 0.0131	2.1992 ± 0.0206	0.3105 ± 0.0064	0.6391 ± 0.0203	0.5603 ± 0.0267
		Yes	0.2179 ± 0.0069	2.2538 ± 0.0108	0.4583 ± 0.0037	0.5776 ± 0.0097	1.0080 ± 0.0154
	2	No	0.0160 ± 0.0016	2.4538 ± 0.0025	0.2861 ± 0.0008	0.1041 ± 0.0030	1.5253 ± 0.0385
		Yes	0.0681 ± 0.0063	2.7813 ± 0.0109	0.1910 ± 0.0028	0.1074 ± 0.0100	0.7072 ± 0.1303
	3	No	1.4801 ± 0.0040	–	–	2.4807 ± 0.0176	4.8695 ± 0.4743
		Yes	0.7986 ± 0.0033	–	–	2.6040 ± 0.0094	4.9530 ± 0.2609
	ε (%)	No	7.1827 ± 0.0310				
		Yes	10.2758 ± 0.0171				
3	1	No	0.1742 ± 0.0076	3.6549 ± 0.0142	0.7242 ± 0.0053	0.8486 ± 0.0141	1.4082 ± 0.0281
		Yes	0.2905 ± 0.0183	4.4777 ± 0.0349	0.4755 ± 0.0099	1.1143 ± 0.0357	0.7544 ± 0.0501
	2	No	0.0885 ± 0.0084	3.3645 ± 0.0150	0.4526 ± 0.0047	0.1987 ± 0.0144	0.9714 ± 0.1228
		Yes	0.0546 ± 0.0041	3.6458 ± 0.0074	0.5561 ± 0.0022	0.1301 ± 0.0063	2.4910 ± 0.0864
	3	No	0.3487 ± 0.0001	–	–	0.9275 ± 0.0012	4.2149 ± 0.0085
		Yes	0.3173 ± 0.0004	–	–	1.4950 ± 0.0064	1.0760 ± 0.0253
	ε (%)	No	1.9123 ± 0.0152				
		Yes	8.6650 ± 0.0106				
17	1	No	0.1484 ± 0.0080	3.3757 ± 0.0151	0.8418 ± 0.0065	0.6389 ± 0.0144	1.8043 ± 0.0455
		Yes	0.0207 ± 0.0021	4.3780 ± 0.0042	0.3579 ± 0.0013	1.1763 ± 0.0069	0.4943 ± 0.0133
	2	No	0.2152 ± 0.0116	2.9225 ± 0.0205	0.5591 ± 0.0074	0.5387 ± 0.0204	0.9941 ± 0.0516
		Yes	0.4705 ± 0.0048	2.8344 ± 0.0085	0.7250 ± 0.0035	0.1470 ± 0.0060	3.4810 ± 0.0704
	3	No	0.8548 ± 0.0004	–	–	0.1702 ± 0.0017	5.1634 ± 0.1110
		Yes	0.8166 ± 0.0001	–	–	0.1426 ± 0.0018	6.6020 ± 0.0852
	ε (%)	No	5.1699 ± 0.0131				
		Yes	8.9515 ± 0.0048				
21	1	No	0.2321 ± 0.0088	6.1282 ± 0.0227	3.2180 ± 0.0189	1.7915 ± 0.0157	7.2733 ± 0.0529
		Yes	0.1136 ± 0.0068	5.5528 ± 0.0200	4.1576 ± 0.0214	1.6702 ± 0.0104	7.9210 ± 0.0451
	2	No	0.0576 ± 0.0002	2.7506 ± 0.0005	0.0628 ± 0.0002	0.0101 ± 0.0001	0.1280 ± 0.0228
		Yes	0.0402 ± 0.0002	2.9211 ± 0.0005	0.0536 ± 0.0002	0.0101 ± 0.0001	0.0990 ± 0.0208
	3	No	0.0188 ± 0.0005	–	–	1.8847 ± 0.0046	5.3105 ± 0.0167
		Yes	0.0161 ± 0.0006	–	–	1.7578 ± 0.0036	4.4671 ± 0.010
	ε (%)	No	7.8681 ± 0.0058				
		Yes	12.3111 ± 0.0049				

When noise is considered, the error in the curve fitting increased mostly by 3.1–6.7%, some can go as high as 12.3%, in comparison with the case without the noise. The influence of segmentation error on the parameters varies from one GB to another, however, the parameters at points 1, 2 are mostly likely affected by the segmentation error, particularly, changes in *c*^1^, $\kappa_{2}^{1}$, $\kappa_{3}^{1}$, $\kappa_{4}^{1}$, *c*^2^, $\kappa_{3}^{2}$, are $\kappa_{4}^{2}$ can be large. Hence, the segmentation error should be reduced as much as possible to improve the accuracy of the inverse estimation. In future, using an automatic segmentation method with small segmentation error as introduced in Bocchi and Rogai (2011) may be the way forward.

In our previous work in Li et al. (2013), the human GB wall was considered to be a non-linear composite material of matrix and two orthogonal families of fibers in the circumferential and longitudinal directions, respectively, and the material parameters were assumed to be constant. These parameters were determined inversely in Li et al. (2013) by using the FEM software-ABAQUS with a user subroutine and a MATLAB code. However, such an inverse approach is extremely time-consuming (~7 h) and unsuitable for clinical applications. In this work, we have developed a simpler approach using analytical or simpler forward solvers, which makes it possible for clinical assessment of in GB human wall disease in real time.

In addition, we extended the previous model from homogenous membrane model in Li et al. (2013) to heterogeneous model, which has significantly improved the fitting accuracy. The heterogeneity of the GB has been confirmed in the experimental work on lamb GB (Genovese et al., 2014). The inverse estimation of the heterogeneous property constants had an error less than 7% for the ten human GB samples, and the computational time was reduced by 20 times (~30 min). Further, allowing the wall thickness variation following experiments (Su, 2005), reduced the error to be less than 4%.

One potential clinical use of the model is to assessing the GB pain. In Table 4, we compare the first principal stress with the pain score associated with the CCK venous injection. It is clear that there is a strong correlation between the magnitude of the stress and the pain score. Although given the limited sample size, the homogeneous and heterogeneous model seem to do equally well in terms of pain prediction.

The limitations of our study should also be mentioned. In the study, the stretches at the points 1–3 were determined analytically during GB emptying phase. The analytical method was based on the GB volume change from images. To our best knowledge, no speckle tracking echocardiography on GB has been reported to validate our model, unlike extensive measured on human left ventricle (Edvardsen et al., 2002; Marwick, 2006; Crosby et al., 2009; Maffessanti et al., 2009; Marwick et al., 2009; Tanaka et al., 2010; Hoit, 2011; Kleijn et al., 2011). In addition, there were also no *in vitro* passive tensile tests on the specimens harvested from the body and fundus of human GB. In future, we may be able to utilize the measured strain/stretch to validate our analytical method for stretch extraction, this will make our regional GB biomechanical property identification more accurate.

Further, we only used one *in vitro* observation to determine the reference configuration of human GBs. In reality, the size of a reference configuration may not be exactly 50% of the size of totally refilled GB. It is possible that the GB reference configuration can be estimated using GB ejection fraction (EF) in cholecystokinin-cholescintigraphy (CCK-CS) (Ozden and DiBaise, 2003) or fatty meal Cholescintigraphy (FM-CS) (Al-Muqbel et al., 2010) exanimation for GB patients' *in vivo* clinical diagnosis.

Although we have investigated the thickness variation in our model, the values we used were applicable only for a healthy GB. When human GBs suffer from diseases such as acute cholecystitis, acalculous cholecystitis and ascites (Sanders, 1980; Runner et al., 2014), the GB thickness can increase significantly. Indeed, diseased GB body wall thickness was ranged in 3–5 mm (Sanders, 1980; Mohammed et al., 2010; Runner et al., 2014). How to estimate the GB wall thickness change in disease will be an important challenge for future studies.

## Conclusions

The heterogeneity of ten samples of human GB is investigated theoretically in refilling phase using a structure-based constitutive model, ellipsoidal GB and membrane in-plane mechanic model. Three different points, two on the equator of GB body with 90° apart and one on the apex of GB fundus, are chosen to evaluate the variation of the material properties. The stretches at these points are tracked over time from the routine ultrasonic images scanned at the Sheffield Hallamshire Hospital during the emptying phase. The material parameters at the three different points are determined inversely using a MATLAB code. The human GBs are found to exhibit heterogeneity, especially from GB body to its apex region. It is found that using a homogeneous model underestimate the peak stresses in the GB wall, and that a strong heterogeneity occurs from GB body to fundus. Finally, our model results indicate that the GB wall is much thicker at the apex, which clarify the contrary findings reported in the literature.

## Author contributions

WL developed math model, designed numerical method, composed and validated code, carried out case studies, analyzed the results and drafted the manuscript. NB checked medical issues in human gallbladder, revised the draft. XL checked the math model, method, result explanation and conducted English text editing.

### Conflict of interest statement

The authors declare that the research was conducted in the absence of any commercial or financial relationships that could be construed as a potential conflict of interest.
